# In vivo reduction of Treg expansion in rodent helminth–malaria coinfection

**DOI:** 10.1186/s13071-026-07465-3

**Published:** 2026-06-13

**Authors:** Luc Bourbon, Aloïs Dusuel, Nicolas Pernet, Mickaël Rialland, Benjamin Roche, Bruno Faivre, Gabriele Sorci

**Affiliations:** 1 Biogéosciences, CNRS UMR 6282, Université Bourgogne Europe, 6 Boulevard Gabriel, 21000 Dijon, France; 2 Center for Translational and Molecular Medicine UMR1231, Université Bourgogne Europe, 21000 Dijon, France; 3 LabEx LipSTIC, 21000 Dijon, France; 4 Plateforme ImaFlow – US58 BioSanD, UFR Sciences de Santé, 7 Boulevard Jeanne d’Arc, 21079 Dijon Cedex, France; 5 MIVEGEC, IRD, CNRS, Université de Montpellier, Montpellier, France

**Keywords:** Excretory–secretory products, Experimental evolution, Helminth immunomodulation, Nematode–malaria coinfection, Regulatory T cells

## Abstract

**Background:**

Parasitic helminths modulate host immunity to establish chronic infections, often expanding regulatory T cells (Tregs) to suppress inflammation. However, how helminths adjust immunomodulation during coinfection with fast-replicating microparasites remains unclear. Here, we investigated the immune modulation dynamics in a murine model coinfected with the nematode *Heligmosomoides polygyrus* (Hp) and the protozoan *Plasmodium yoelii* (Py).

**Methods:**

We compared Treg expansion in single Hp infected and coinfected mice, assessed Hp excretory–secretory products (HES) for Treg induction in vitro, measured expression of the TGF-β mimic *Hp-TGM-1* gene, and conducted experimental evolution over eight generations in single and coinfected hosts.

**Results:**

Although coinfected mice exhibited reduced Treg expansion in vivo, HES collected from worms in single infected and coinfected hosts induced similar Treg expansion in vitro. Furthermore, *Hp-TGM-1* expression was found to be upregulated in worms from coinfected hosts. Experimental evolution revealed no consistent differences between Hp lines (i.e., parasites maintained in single infected or coinfected hosts).

**Conclusions:**

These results suggest that reduced Treg expansion during coinfection is driven by the host rather than the parasite, which informs our understanding of immune modulation in helminth–malaria coinfections.

**Graphical abstract:**

**Figure:**
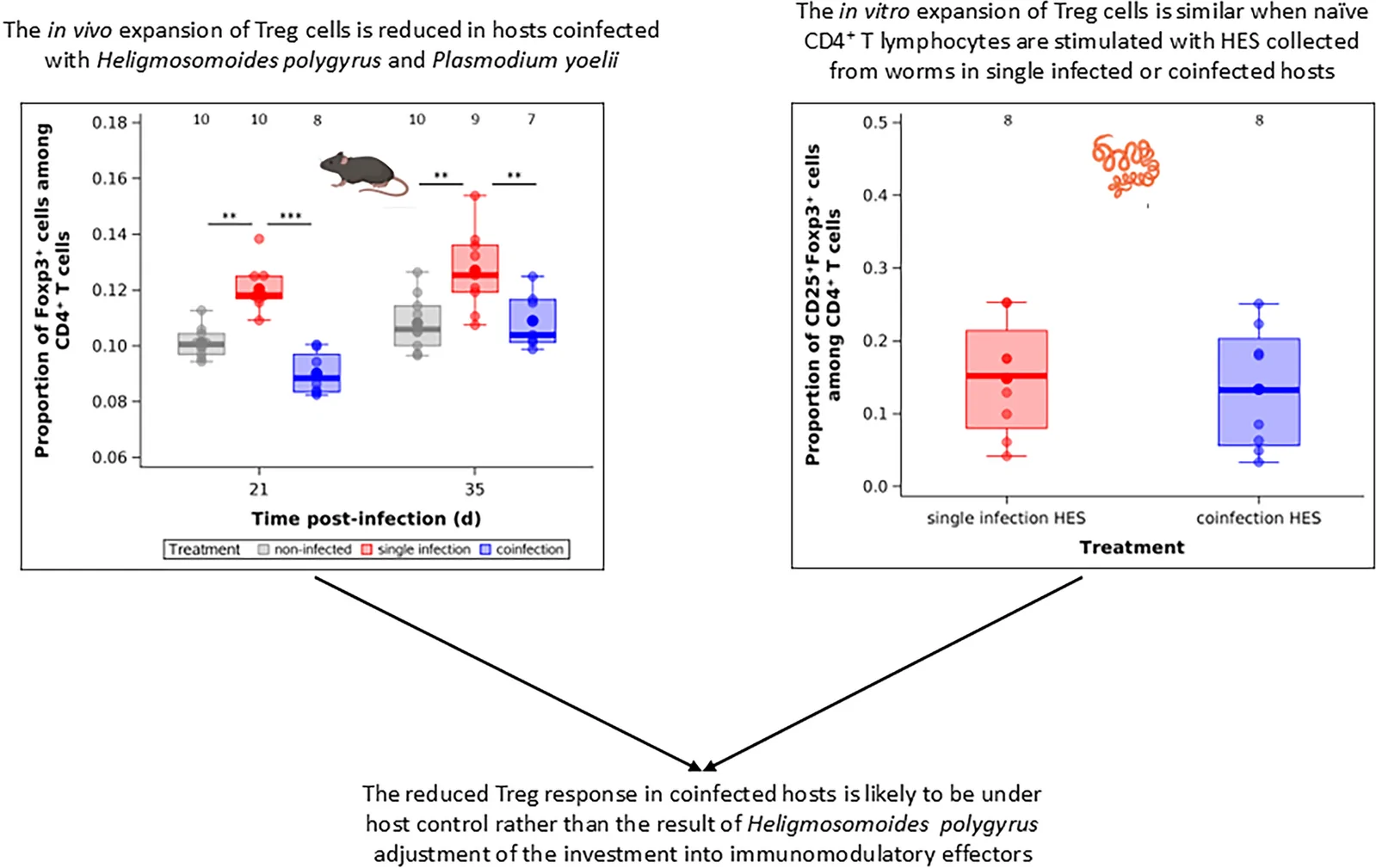

**Supplementary Information:**

The online version contains supplementary material available at 10.1186/s13071-026-07465-3.

## Background

Parasitic helminths are complex metazoans that can elicit strong immune responses owing to their relatively large size and the tissue damage they cause during migration from the entry site (e.g., the skin) to their final infection site (e.g., the gut) [1–4]. Despite this, helminths often establish chronic infections that can persist for months or even years [5, 6]. To achieve long-term persistence despite host immunity, helminths have evolved sophisticated immune regulatory mechanisms [7, 8], including masking of immune epitopes and direct interference with host immune responses via immunomodulatory molecules [9]. These immune interference strategies are thought to be adaptive, as they allow helminths to increase their lifetime production of propagules, by extending the patent period (the duration of infection during which the parasite produces offspring). However, hosts are frequently coinfected with multiple parasites that can have different life cycles and potentially conflicting interests [10–12].

Coinfection between helminths and microparasites is a widespread phenomenon in humans, livestock, and wildlife and has the potential to affect both the severity of the disease symptoms for the host and the dynamics of the coinfecting parasites [10, 13–15]. The within-host interactions between helminths and microparasites can be driven by different mechanisms, involving competition for common resources (for instance, between hematophagous helminths and *Plasmodium*) or reciprocal antagonistic effects between anti-helminthic and anti-microbial immune effectors [11, 16]. These immune-mediated interactions have been well-documented in several systems, involving different helminth and microparasite species (including viruses) [17–22]. For instance, mice coinfected with the nematode *Heligmosomoides polygyrus* and the West Nile virus suffered from increased mortality, mediated by a Th2-skewed immune response [18, 19]. Several studies have reported the effect of helminths on the infection dynamics of microparasites that do not share the same site of infection. Infection with SARS-CoV-2 can produce severe symptoms often mediated by a pro-inflammatory cytokine storm, but concomitant infection with the nematode *Trichinella spiralis* has been shown to protect against TNF-α and IFN-γ shock in mice, through an IL-9-dependent mechanism [23]. Similarly, helminth infection can interfere with the dynamics of both pulmonary and extrapulmonary tuberculosis, again through the dual effect of Th2 and regulatory cytokines [24, 25], while infection with the nematode *Trichuris muris* produced an increased pneumococcal carriage density [26]. In the same line, using a coinfection model involving the nematode *Heligmosomoides polygyrus* and the apicomplexan *Plasmodium yoelii*, we have shown that coinfection exacerbates the symptoms of malaria in an order-dependent way, and that the increased disease severity is mediated by a Th2 skewed immune response [27].

This nonexhaustive review suggests that the immune interactions between helminths and microparasites in coinfected hosts have the potential to affect the infection dynamics and the transmission success of both infectious agents. In particular, if helminth-induced immunoregulation increases the risk for the host to be overwhelmed by fast-replicating microparasites [18, 19, 28–33] and to succumb to the infection [34], the fitness of the helminth may be compromised owing to a reduced infectious period and an overall lower inter-host transmission. Therefore, while immune modulation may be adaptive in single infections, in coinfected hosts, helminths may need to adjust their investment in immunomodulatory molecules to balance the benefits of reduced immune-mediated mortality and fecundity impairment against the risk of host death from microparasite overgrowth. This suggests that the optimal level of immune interference for helminths may shift in the context of coinfection. Previous work has shown that helminth-induced immunomodulation can vary between infection stages, parasite genotypes, and host context, supporting the view that helminths can adjust their level of immune modulation [35–38]. However, to the best of our knowledge there is no work that has explicitly investigated whether helminths can adaptively respond to microparasite coinfection by up/downregulating their level of immunomodulation.

Here, we wished to explore if helminths can adjust their level of immunomodulation using a murine model of coinfection between the gastrointestinal nematode *Heligmosomoides polygyrus* (hereafter, Hp) and the protozoan *Plasmodium yoelii* (hereafter, Py). Hp is a model system in immunoparasitology [39, 40], and its capacity to modulate the host immune response has been extensively studied [41–48]. In mice, Hp infection induces a tolerogenic immune phenotype, characterized by an expansion of CD25^+^Foxp3^+^ regulatory T cells (Tregs), orchestrated by regulatory cytokines such as TGF-β [46, 49–51]. This tolerogenic state refers to an immune environment that is less likely to mount damaging inflammatory responses. As a result, Hp infection has been shown to protect hosts against inflammatory and immune-mediated diseases [52–59]. Mechanistically, this immune phenotype is thought to be driven by Hp excretory/secretory products (HES) [60–67]. In particular, Hp secretes molecules (Hp-TGM), that mimic the host cytokine TGF-β and can induce the expansion of Tregs both in vitro and in vivo [68–70], replicating the effect of TGF-β on CD4^+^ T cells [71] and suppressing allergic responses [72].

In this study, we first compared the expansion of the population of Treg cells in vivo in mice infected with Hp alone versus those coinfected with Hp and Py, predicting that coinfection would result in a weaker expansion of Treg cells. However, since Treg expansion in vivo is influenced by both host and parasite factors, observing a reduced Treg response in coinfected mice does not conclusively demonstrate that Hp adjusts its investment in immunomodulation. To address this, we also examined two mechanisms that are solely under Hp control: (1) the ability of HES to induce expansion of Treg cells in vitro, and (2) the gene expression of the TGF-β mimic *Hp-TGM-1*. We hypothesized that HES collected from worms in coinfected hosts would induce a weaker expansion of Treg cells in vitro, and that these worms would exhibit downregulated expression of the *Hp-TGM-1* gene. This approach allowed us to test whether Hp can plastically (i.e., within-generation) adjust its investment in immunomodulatory effectors in response to the environment provided by a coinfected host.

In addition to investigating the phenotypic plasticity of Hp investment in immunomodulatory molecules, we conducted a second experiment to determine whether Hp could adapt to the environmental conditions provided by a coinfected host through a microevolutionary response (i.e., changes occurring over multiple generations). To achieve this, we serially passaged Hp through two host environments: single-infected mice (SI-lines) and mice that had previously been infected with Py (COI-lines). After eight generations of experimental evolution, we used worms from both lines to infect naïve hosts in single infection trials. This allowed us to compare their capacity to modulate the host immune response with that of the ancestral Hp population. We predicted that Hp from the COI-lines would show reduced investment in immunomodulatory effectors after eight generations of selection.

Finally, in a third experiment, we assessed whether within-host persistence and cumulative egg excretion differed between the evolved lines and the ancestral Hp population (and between SI-lines and COI-lines) during single infection trials. This experiment enabled us to investigate the extent to which consistent exposure to the conditions provided by a coinfected host affected two key traits that describe the infection dynamics of Hp.

### Experimental animals and infections

C57BL/6JRj female mice (7–10 weeks old) were purchased from Janvier Labs (Le Genest-Saint-Isle, France), housed in cages containing five individuals under pathogen-free conditions, and maintained under a constant temperature of 24 °C and a photoperiod of 12h:12h light:dark with ad libitum access to water and standard chow diet (A03-10, Safe, Augny, France). All mice were acclimatized to the housing conditions for (at least) 7 days prior to the start of the experiments, were monitored twice a day to check health status, and euthanized by cervical dislocation under anesthesia with isoflurane at specific days post-infection (p.i.) for terminal collection of organs.

Mice were infected with *Heligmosomoides polygyrus bakeri* by oral gavage with 350 L3 larvae suspended in 0.2 ml of drinking water and with *Plasmodium yoelii* 17XNL by intraperitoneal injection of 5 × 10^5^ infected red blood cells (iRBC) suspended in 0.1 ml PBS. To obtain L3 larvae, feces from infected mice were mixed with distilled water and charcoal, then spread on Whatman paper placed on a wet paper towel in a Petri dish. The dishes were kept at room temperature in the dark, with regular monitoring of humidity. After 11 days, L3 larvae were collected by washing the underside of the Whatman paper, the paper towel, and the bottom of the Petri dish with distilled water. The larvae were then isolated by centrifugation (100 rpm, 10 min, 4 °C), washed twice, and stored at 4 °C in distilled water until use. Py was kindly provided by Dr. Sarah Reece (University of Edinburgh) and cryopreserved in liquid nitrogen. Prior to the experimental infections, Py was revitalized by intra-peritoneally injecting 100 µl of the cryopreserved blood into a mouse. At day 14 p.i., we collected 10 µl of blood to assess the red blood cell count using a SCIL Vet abc Plus + hematology analyzer (Horiba Medical, Montpellier, France), and a drop of blood was smeared on a slide to assess parasitemia. Slides were fixed with methanol and then stained with a Giemsa RS solution (Carlo Erba Reagents, Val de Reuil, France) (10% v/v in phosphate buffer) for 15 min and rinsed with water. Infected red blood cells were counted using an optical microscope (Eclipse E600, Nikon Corporation, Tokyo, Japan) under magnification (1000×). Knowing the red blood cell count and the proportion of infected red blood cells, we could quantify the volume of blood needed to have a dose of 5 × 10^5^ infected red blood cells suspended in 0.1 ml PBS.

The choice of Hp and Py as coinfecting parasites was motivated by our previous knowledge of the system (e.g., refs. [27, 73, 74]), and the fact that it meets the assumptions underlying the hypothesis under investigation (e.g., immunomodulation, the dual effect between anti-helminthic and anti-malaria immune responses, and the conflict between a fast-replicating microparasite and a helminth with a long patent period). However, other microparasite/helminth associations should also be suitable to investigate if and how coinfection alters the investment into the helminth immunomodulation strategy.

#### Experiment 1: within-generation effect of coinfection on the investment into immunomodulation

Mice were randomly assigned to three experimental groups. A control group of mice (*n* = 20), used for the assessment of Treg cells in vivo, was sham infected (intraperitoneal injection of 0.1 ml of PBS and oral gavage with a 0.2 ml of drinking water), one group (*n* = 29) received a single Hp infection, and one group (*n* = 25) was infected with Py and 14 days after with Hp (day 14 post-Py infection corresponding approximately to the peak of the acute infection). Infected mice were euthanized, as described above, at day 14 post-Hp infection (*n* = 10 per group) to retrieve adult worms for the collection of HES; the remaining mice were euthanized at day 21 and 35 post-Hp infection (control: *n* = 10 and 10, single infection: *n* = 10 and 9, and coinfection: *n* = 8 and 7, respectively) to collect host spleen for the assessment of Treg cells in vivo and adult worms for the analysis of *Hp-TGM-1* gene expression. The experiment was run in two blocks.

#### Experiment 2: effect of experimental evolution in single infected or coinfected hosts on the investment into immunomodulation

**Fig. 1:**
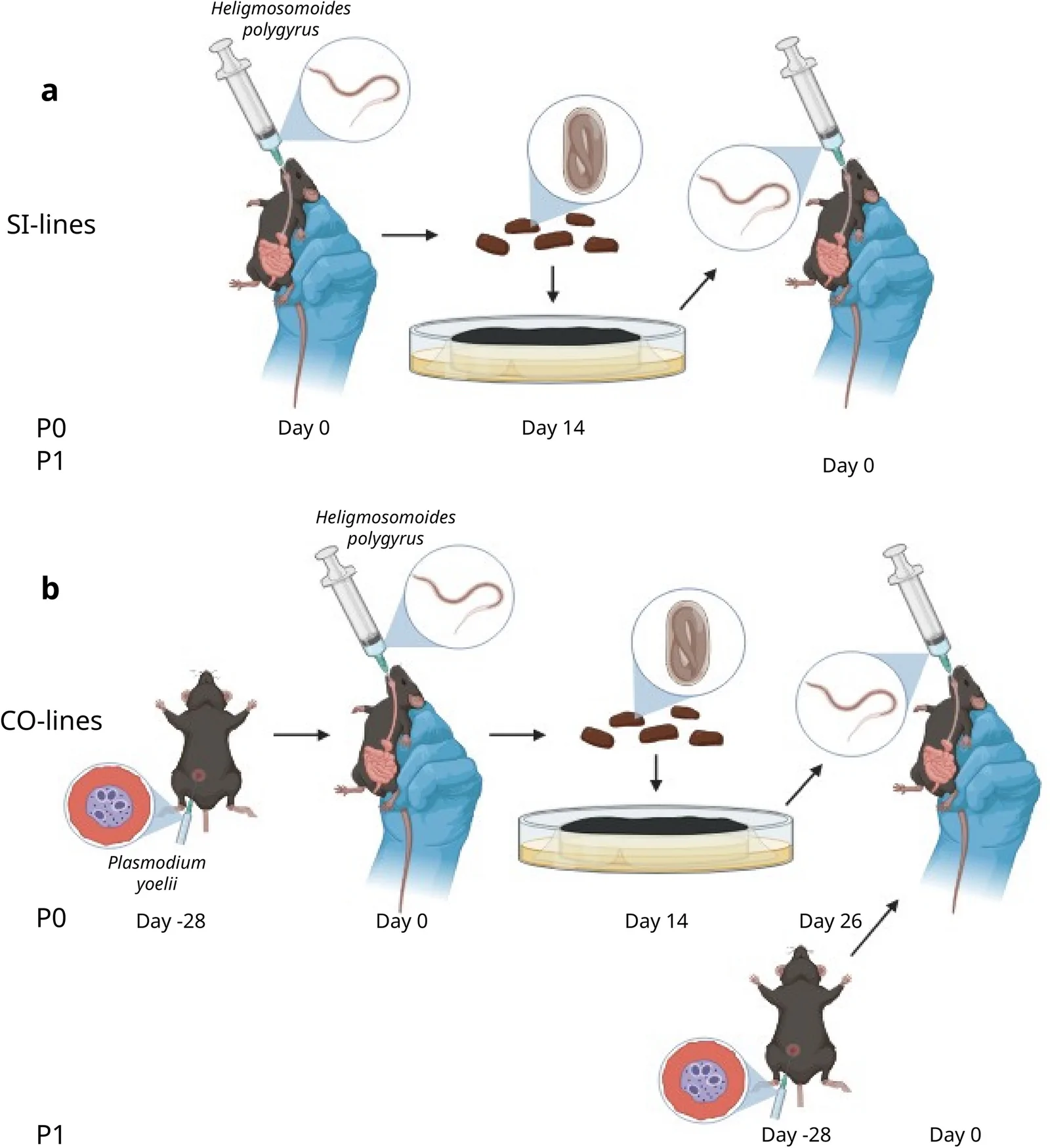
Experimental design used for the serial passages of Hp in **a** single and **b** coinfected mice. **a** At day 0, five mice were infected with Hp L3 larvae by gavage. At day 14 p.i., feces were collected and cultured to harvest L3 larvae that were used to infect a new batch of five naïve mice (one mouse contributing larvae to infect the subsequent mouse). This sequence was repeated for eight passages (with five replicated Hp lines). **b** For the passages in coinfected hosts, the experimental design was the same, with the exception that mice were first infected with Py, and at day 14 post-Py infection, they were infected with Hp. Only Hp was transmitted from host to host, while Py came from our cryopreserved stock. Figure elements were created with BioRender

We conducted serial passages of Hp either in single infected or in coinfected mice. For the single-infected lines (SI-lines), mice were infected with Hp (from our stock population, hereafter named ancestral). At day 14 p.i., feces were collected and cultured to harvest L3 larvae that were then used to infect a new batch of mice. This sequence was repeated for eight passages. Five mice were infected per passage (one mouse contributing the larvae to infect the subsequent mouse), providing five replicated lines of worms; however, one line was lost because we could not culture enough larvae to allow the inter-host transmission, reducing the number of replicated lines to four. For the coinfection lines (COI-lines), mice were first infected with Py (from our stock population) and, at day 14 post-Py infection, they were infected with Hp. At day 14 post-Hp infection, feces were collected and cultured to harvest L3 larvae that were used to infect a new batch of mice that had been previously infected with Py (14 days prior to the Hp infection). This sequence was also repeated for eight passages. At each passage, only Hp was serially transferred from host to host, while Py always came from the initial stock population. Therefore, only Hp was allowed to evolve. As for the SI-lines, five mice were infected per passage, and one line was lost for the same reason. The number of passages was determined on the basis of previous work showing that serial passage of Hp produced microevolutionary responses in less than ten passages [35, 75], although this might also depend on the trait under investigation. Figure 1 gives a schematic illustration of the design used for the experimental evolution of Hp.

After these eight passages, feces of mice infected with SI-lines or COI-lines were collected and cultured to harvest L3 larvae. These larvae were used to infect mice (in single infection trials) which allowed us to compare the capacity to interfere with the host immune response of the evolved lines to the ancestral Hp population. In total, 30 mice were infected with Hp from SI-lines and 30 mice with Hp from COI-lines in two blocks. Mice were euthanized as described above at day 14 p.i. (*n* = 10 for SI-lines and 10 for COI-lines) to retrieve adult worms for the collection of HES products; the remaining mice were euthanized at days 21 and 35 p.i. to collect host spleens for the assessment of Treg cells in vivo and adult worms for the analysis of *Hp-TGM-1* gene expression.

#### Experiment 3: effect of experimental evolution in single infected or coinfected hosts on within-host persistence and cumulative egg excretion

In total, 30 mice were infected with Hp from the ancestral population, with Hp from the SI-lines or with Hp from the COI-lines (10 mice per group), in single infection trials. These mice were monitored up to 99 days p.i. to assess the effect of coinfection on Hp within-host persistence and the cumulative egg shedding. Excretion of Hp eggs in host feces was assessed 14 times between day 12 and day 99 p.i., as described in the section Assessing Hp egg excretion. We considered that mice that shed no eggs during at least two consecutive sampling dates had cleared the infection.

### Assessment of Foxp3^+^ Treg cells in vivo

Spleens were homogenized with a 70-µm cell strainer and washed two times with PBS to obtain a single-cell suspension. Red blood cells were removed from suspensions with eBioscience™ RBC Lysis Buffer (Invitrogen™) (3 min incubation at room temperature) and washed two times with PBS before flow cytometry analysis.

### Hp excretory–secretory products

Collection of adult worms and purification of HES were performed as described by Johnston et al. [76]. Briefly, mice were euthanized by cervical dislocation under isoflurane anesthesia and the intestine was immediately removed. Adult worms were collected by scraping the duodenal lumen, separated from gut debris by migration through a muslin bag (Baermann apparatus), and then washed with pyrogen-free PBS several times. Adult worms from five infected mice were pooled together and maintained at 37°C/5% CO_2_ in T25 flasks containing 10 ml of RPMI 1640 (1.2% glucose, 3 mM l-glutamine, 5 U/ml penicillin, and 5 µg/ml streptomycin) placed upright. The batch of HES-containing medium from the first 24 h of culture was discarded to avoid any contamination, and a second batch was collected 3 days later (4 days after adult worm harvest) and stored at −80 °C.

HES-containing medium was concentrated/washed three times with 10 ml of pyrogen-free PBS using a centrifugal 3-kDa cut-off concentrator (Amicon^®^ Ultra 15 ml, Merck Millipore, Darmstadt, Germany). HES was concentrated down to a volume of approximately 1 ml, sterile-filtered through 0.22-µm low-protein binding filters, and aliquoted for storage at −80 °C. Total protein concentration was measured with a Pierce™ BCA Protein Assay Kit (Thermo Scientific™, Waltham, Massachusetts, USA).

### Naïve CD4^+^ T-cell enrichment and stimulation with HES in vitro

Spleen, axillary, brachial, cervical, and inguinal lymph nodes were collected from naïve C57BL/6JRj mice, pooled and homogenized with a 70-µm cell strainer, and washed with RPMI 1640. Naïve CD4^+^ T cells were negatively selected from single cell suspensions using a mouse Naive CD4^+^ T Cell Isolation Kit (Miltenyi Biotec, Bergisch Gladbach, Germany) according to the manufacturer’s instructions. Then, 96-well flat-bottom culture plates were coated with 5 µg/ml anti-CD3 (145-2C11 clone, Bio X Cell, Lebanon, New Hampshire, USA) and 2 µg/ml anti-CD28 (PV-1 clone, Bio X Cell) in 100 µl of PBS for 2 h at 37 °C.

The antibody coating mix was gently discarded from the culture plate, and naive CD4^+^ T cells were cultured at 150k cells/well in 200 µl of complete medium [RPMI 1640, 10% fetal bovine serum (FBS), 10 mM HEPES buffer, 2 mM l-glutamine, 55 µM 2-ME, 1 mM sodium pyruvate, MEM NEAA (non-essential amino acids diluted from 100× ready to use solution), 100 U/ml penicillin, 250 ng/ml amphotericin B, and 100 µg/ml streptomycin] for 72 h in the presence of HES (10 ug/ml total protein) at 37°C/5% CO_2_. As a control, CD4^+^ T cells were cultured with HES collected from worms in single infected hosts that were heat-inactivated at 80 °C for 30 min. In an additional control, HES products were replaced by PBS. Cells were then treated with BD GolgiStop™ Protein Transport Inhibitor (BD Biosciences, Franklin Lakes, New Jersey, USA) and eBioscience™ Cell Stimulation Cocktail (Invitrogen™) in complete RPMI medium for 4 h at 37°C/5% CO_2_, and washed two times with PBS before flow cytometry analysis for the assessment of Foxp3^+^ T cells.

### Flow cytometry

Live cells were counted with viability trypan blue dye and dispatched in 96-well V-bottom plates to obtain 1.10^7^ cells/well for splenocytes (in vivo experiment, infected mice) and 1.10^5^ cells/well for CD4^+^ T cells (in vitro experiment, stimulation with HES). Cells were then stained with LIVE/DEAD™ Fixable Blue Dead Cell Stain Kit (Invitrogen) in PBS for 30 min at 4 °C, incubated in stain buffer with BD Fc Block™ (BD Pharmingen, Franklin Lakes, New Jersey, USA) for 15 min at room temperature (0.5 µg/well), and stained with surface antibodies in brilliant stain buffer (see Table S1 for details on the markers used for each experiment) for 30 min at 4 °C. Cells were then fixed and permeabilized with eBioscience™ Foxp3/Transcription Factor Staining Buffer Set (Invitrogen) prior to intracellular staining in brilliant stain buffer (Table S1) for 30 min at room temperature. Cells were resuspended in stain buffer, filtered with a 100-µm nylon mesh, and analyzed on a 4-laser Cytek Aurora™ spectral flow cytometer (Cytek^®^ Biosciences, Fremont, California, USA). Events were gated and analyzed with SpectroFlo^®^ v3.0.0 software (Cytek® Biosciences) (see Figs. S1 and S2 for the gating strategies for the in vivo and in vitro experiments, respectively).

### Reverse transcription quantitative PCR (RT-qPCR) for *Hp-TGM-1* gene expression

Mice were euthanized by cervical dislocation under isoflurane anesthesia; intestines were immediately removed, longitudinally opened, and worms were collected and stored in RNAlater™ solution (Invitrogen, Carlsbad, California, USA) at −80 °C until use. Worms were homogenized in TRIzol™ Reagent (Invitrogen) under strong agitation using 0.5-mm glass beads and Precellys^®^ 24 Touch homogenizer (Bertin Technologies, Montigny-le-Bretonneux, France). RNA extraction was performed following the manufacturer’s instructions. RNA concentration was measured with NanoPhotometer^®^ N50 (Implen, Munich, Germany). Reverse transcription was performed with the High-Capacity cDNA Reverse Transcription Kit (Applied Biosystems, Foster City, California, USA) from 0.5 µg total RNA. Quantitative PCR were performed with PowerUp™ SYBR™ Green Master Mix (Applied Biosystems) on a QuantStudio™ 3 Real-Time PCR System (Applied Biosystems). We used *β-actin* as the reference gene (see Table S2 for primer sequences), and quantified *Hp-TGM-1* expression as the ΔCt with respect to *β-actin*.

### Assessing Hp egg excretion

To assess egg excretion (number of parasite eggs per gram of host feces), mice were individually placed for 1 h in clean cages; 200 mg of feces were then collected and disposed in tubes containing 2.5 ml of water. Feces were mechanically broken up and 5 ml of salted water (75% saturation, 0.27g NaCl per ml of water) was added to allow egg flotation. Eggs were counted in a McMaster chamber using an optical microscope (Eclipse E400, Nikon, Tokyo, Japan) under magnification (150×).

### Statistical analyses

#### (a) Experiment 1

The effect of the treatment (single Hp infection or coinfection) on the expansion of Treg cells in vivo was assessed using a generalized linear model with a beta-distribution of errors, as the response variable was the proportion of Foxp3^+^ cells among CD4^+^ T lymphocytes. The explanatory variables were the experimental group (single Hp infection or coinfection), the time p.i. (day 21 or 35), and the two-way interaction.

The effect of coinfection on the capacity of HES to induce an expansion of Treg cells and GATA-3^+^ cells in vitro (the proportion of Foxp3^+^ cells and the proportion of GATA-3^+^ cells among CD4^+^ T lymphocytes) was assessed using generalized linear mixed models with a beta-distribution of errors. We first ran models to investigate whether the capacity to induce an expansion of Treg and GATA-3^+^ cells differed between HES collected from worms in single-infected hosts and the two control treatments (PBS and heat-inactivated HES). These in vitro tests were repeated using four different donor mice (mice that provided the spleen, axillary, brachial, cervical, and inguinal lymph nodes), therefore the models also included the identity of the donor mouse as a random intercept. We then ran models where the capacity of HES to induce an expansion of Treg and GATA-3^+^ cells was compared between HES collected from worms in single-infected and coinfected hosts. The test was repeated using eight different donor mice and, as above, the identity of the donor mouse was included as a random effect in the model. In all models, we also included the proportion of CD4^+^IFN-γ^+^ cells as a covariate, to control for a possible effect of contamination of the HES solution.

The effect of coinfection on the expression of *Hp-TGM-1* gene (ΔCt with *β-actin* as the reference gene) was assessed using a two-way analysis of variance (ANOVA) with experimental group (single-infection or coinfection), time p.i. (day 21 or 35), and the two-way interaction as explanatory variables. We also computed the log_2_ fold change of *Hp-TGM-1* for worms in coinfected hosts (the worms in single-infected hosts being the reference group), to visualize the magnitude of the effect.

#### (b) Experiment 2

The effect of serial passage on the expansion of the population of Treg cells in vivo was assessed following a two-step procedure. First, we ran a generalized linear model with a beta-distribution of errors where the proportion of Tregs was the response variable and the treatment (ancestral, SI-lines, COI-lines), the time p.i. (day 21 or 35), and the two-way interaction were the explanatory variables. Since we could not include the replicated Hp line ID in this model, we ran a linear mixed model where the response variable was the logit-ratio between the proportion of Tregs in mice infected with SI-lines or COI-lines and the mean proportion of Tregs in mice infected with the ancestral population: [logit (Treg_evolved_) – logit (mean_ancestral_)]. Positive logit-ratio values indicate a higher proportion of Tregs in mice infected with the evolved lines compared with mice infected with the ancestral population, while negative values indicate a lower proportion. The model included the treatment (SI-lines or COI-lines), the time p.i. (day 21 or 35), and the two-way interaction as fixed effects. The replicated line ID was included as a random effect. Moreover, to deal with the uncertainty around the estimate of the mean proportion of Tregs for the ancestral population, we computed the scaled weights as the inverse of the variance of the logit-ratios divided by the mean weight, and included them in the model.

The effect of serial passage on the expansion of the population of Treg and GATA-3^+^ cells in vitro was assessed using generalized linear mixed models with a beta-distribution of errors. The proportion of Treg or GATA-3^+^ cells was the response variable, while the treatment (ancestral, SI-lines, COI-lines) was included as a fixed effect. The identity of the donor mouse was declared as a random factor, but not the identity of the replicated Hp lines, because the worms from the different replicated lines were pooled together to maximize the amount of HES collected. The proportion of CD4^+^IFN-γ^+^ cells was included as a covariate, to control for a possible effect of contamination of the HES solution.

For the expression of the *Hp-TGM-1* gene, we computed the ΔΔCt as the difference between the ΔCt values of the evolved lines and the mean ΔCt of the ancestral population; 0 indicating no difference between the ancestral and the evolved lines. We first ran an intercept-only model with the aim of investigating whether ΔΔCt values were significantly different from 0 (i.e., 95% confidence intervals of the intercept nonoverlapping 0), which would indicate that the evolved lines had a significantly different gene expression compared with the ancestral population. We then ran a model that included the treatment (SI-lines, COI-lines), time p.i. (day 21, 35), and the two-way interaction as fixed effects. The identity of the replicated Hp lines was declared as a random factor. As for the model on the expansion of the Treg cells in vivo, we computed the variance of the ΔΔCt to handle the uncertainty around the mean ΔCt of the ancestral population, and included the scaled weight (as computed above) in the model.

#### (c) Experiment 3

The within-host persistence of the Hp infection was analyzed using the Kaplan–Meier estimator, and significance was assessed using a log-rank test. For the analysis of cumulative egg shedding, we added the number of eggs excreted during each of 14 sampling dates (from day 12 to day 99 p.i.) for each mouse in each experimental group. These values were ln-transformed and analyzed using a one-way ANOVA with treatment (ancestral, SI-lines, COI-lines) as the explanatory variable.

For all experiments, we also conducted post hoc comparisons of least-squares means and *P*-values were adjusted for multiple tests using the Bonferroni method. The full model outputs are reported in tables with the parameter estimates and the 95% confidence intervals, whereas the post hoc comparisons are reported in the figure legends.

All the statistical analyses and figures were done using SAS Studio.

## Results

### Experiment 1: within-generation effect of coinfection on the investment into immunomodulation

**Fig. 2:**
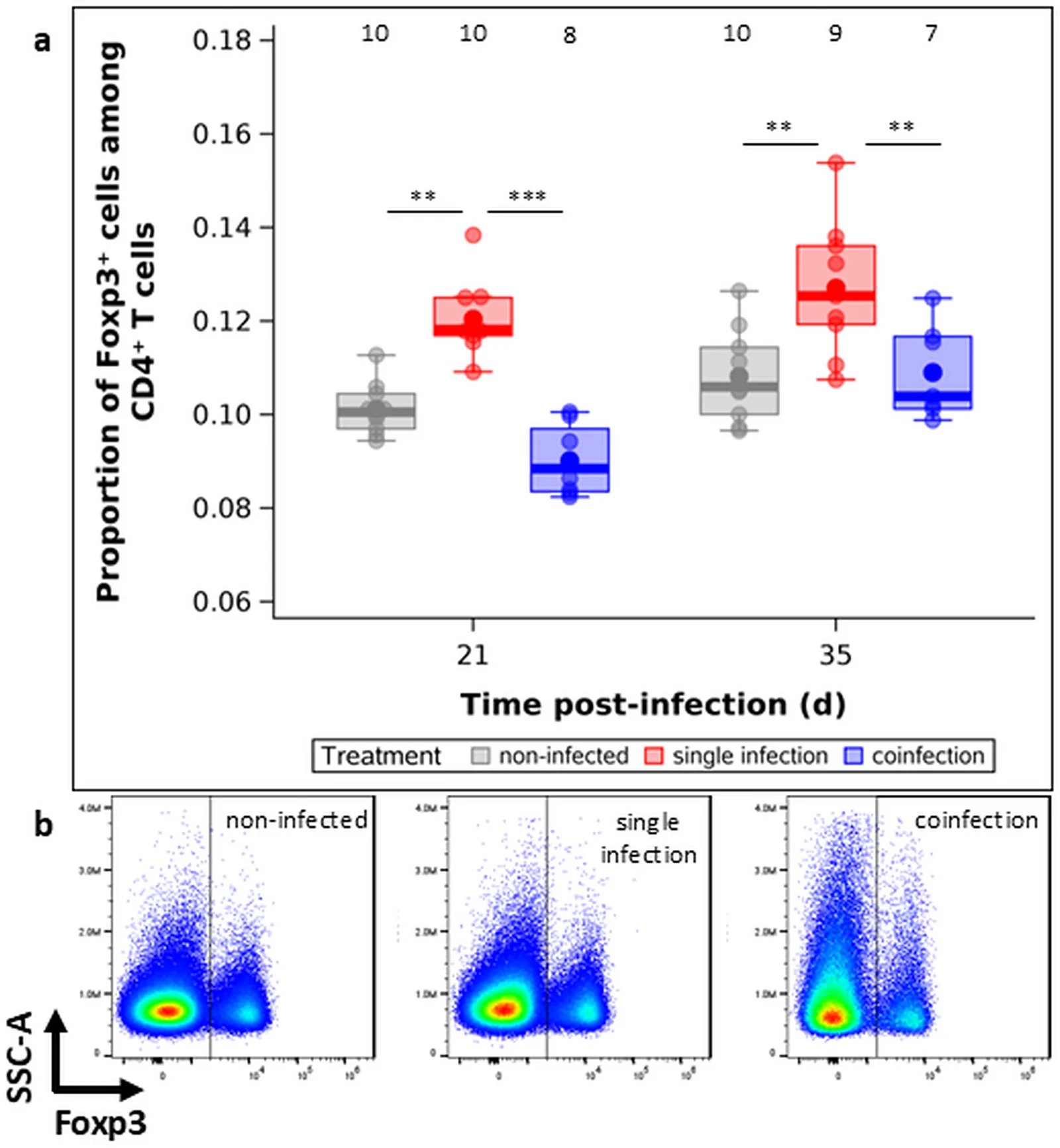
Expansion of Treg cells in vivo. **a** Proportion of Foxp3^+^ cells among CD4^+^ T lymphocytes in spleens of noninfected, single-Hp infected and coinfected mice, at days 21 and 35 post-infection. The proportion of Tregs in the single infection group was significantly higher than in the noninfected group at both times p.i. (Bonferroni adjusted *P* = 0.0002 and *P* = 0.0006, respectively). The proportion of Tregs in the coinfection group was significantly lower than in the single infection group at both times p.i. (Bonferroni adjusted *P* < 0.0001 and *P* = 0.0045, respectively). The proportion of Tregs in the coinfection group did not differ from the noninfected group at any time p.i. (Bonferroni adjusted *P* = 0.0946 and *P* = 1, respectively). Dots represent individual observations, boxes represent the interquartile range (IQR), the horizontal line within each box indicates the median, the larger dot indicates the mean, whiskers extend to the most extreme values within 1.5 × IQR from the quartiles, and points beyond the whiskers indicate outliers. Numbers above the boxes indicate the sample size. **b** Representative biaxial flow cytometry plots (one per experimental group) displaying intracellular Foxp3 versus side scatter area (SSC-A) in CD4^+^ T cells (see Fig. S1 for gating strategy)

We first investigated whether coinfection affected the expansion of the population of Treg cells in vivo. Using a generalized linear model with a beta-distribution of errors, we found that single infected mice had a higher proportion of Treg cells compared with control, noninfected mice at both day 21 and 35 p.i. (see Fig. 2 for post hoc pairwise comparisons). In contrast, coinfection significantly reduced the proportion of Treg cells compared with single infected hosts at both sampling days (see Fig. 2 for post hoc pairwise comparisons). The differences between groups were more pronounced at day 21 p.i. than at day 35 p.i., as indicated by the marginally significant interaction between treatment and time p.i. (Table 1).

**Table 1:** Generalized linear model with a beta-distribution of errors exploring the effect of treatment (noninfected, single-Hp infection, coinfection) on the expansion of Treg cells in vivo at days 21 and 35 post-Hp infection

Effects	*df*	*F*	*P*
Treatment	2, 48	38.09	< 0.0001
Time p.i.	1, 48	20.99	< 0.0001
Treatment × time p.i.	2, 48	3.43	0.0406

We report the degrees of freedom (*df*), *F*, and *P* values. *N* = 54

**Fig. 3:**
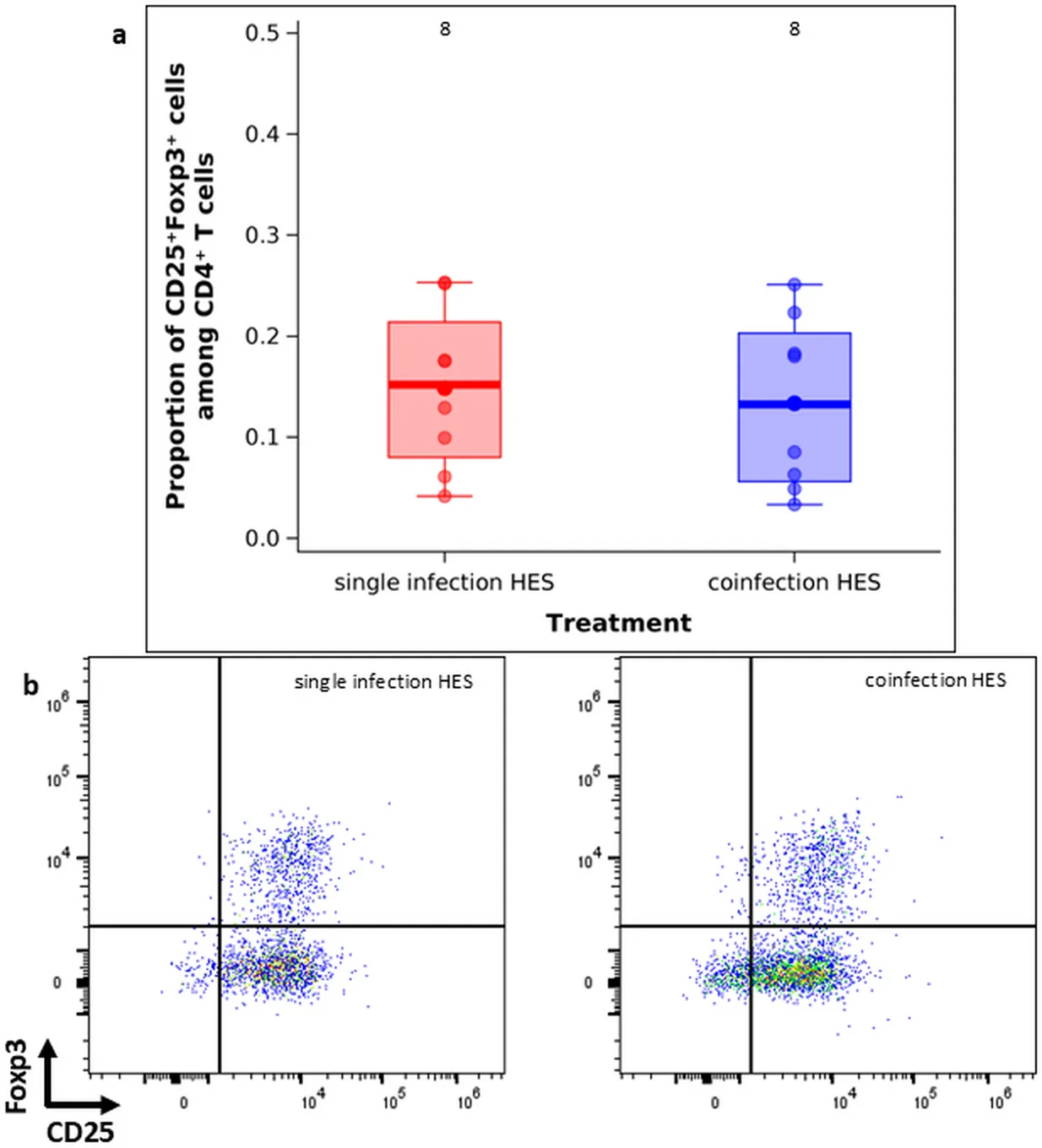
In vitro differentiation of naïve CD4^+^ T lymphocytes into CD25^+^Foxp3^+^ cells. **a** Proportion of CD25^+^Foxp3^+^ cells after in vitro exposure of naïve CD4^+^ T lymphocytes to HES collected from worms in single-infected mice or from worms in coinfected mice. The proportion of Treg cells did not differ between the single-infection HES and the coinfection HES (*P* = 0.9320). Dots represent individual observations, boxes represent the interquartile range (IQR), the horizontal line within each box indicates the median, the larger dot indicates the mean, whiskers extend to the most extreme values within 1.5 × IQR from the quartiles, and points beyond the whiskers indicate outliers. Numbers above the boxes indicate the sample size. **b** Representative biaxial flow cytometry plots (one per experimental group) displaying intracellular Foxp3 versus surface CD25 expression within the CD4^+^ T-cell population (see Fig. S2 for the gating strategy)

We then investigated whether this difference could be due to mechanisms solely under Hp control. To do this, we verified whether the previous results could be reproduced in vitro when naïve CD4^+^ T cells were stimulated with HES collected from worms in single infected or coinfected hosts. We first ran a generalized linear mixed model with a beta-distribution of errors where we compared the differentiation of naïve CD4^+^ T lymphocytes into CD25^+^Foxp3^+^ cells when stimulated with HES collected from worms in single-Hp infected hosts, with heat-inactivated HES collected from worms in single-infected hosts or with PBS. As expected, we found that HES from worms in single-Hp infected hosts induced an expansion of Treg cells in vitro compared with both control groups (Table S3; Fig. S3a). When focusing on the population of GATA-3^+^ cells, there was no difference between the groups, with virtually no differentiation into GATA-3^+^ cells (Table S4; Fig. S3b). We subsequently ran another generalized linear mixed model where we compared the capacity of HES collected from worms in single and coinfected mice to differentially expand the population of Treg and GATA-3^+^ cells. HES from the two treatment groups induced a similar expansion of Treg cells (Table 2; Fig. 3), and still virtually no differentiation into GATA-3^+^ cells (Table S5; Fig. S4).

**Table 2:** Generalized linear mixed model with a beta-distribution of errors exploring the effect of treatment (single-Hp infection, coinfection) on the capacity of HES to expand the population of CD25^+^Foxp3^+^ cells in vitro

Fixed effects	Estimate	95% CI	*df*	*F*	*P*
Treatment			1, 6	0.01	0.9320
Single-Hp infection	0				
Coinfection	0.017	0.460/0.494			
Proportion of CD4^+^IFN-γ^+^ cells	−21.057	−36.749/−5.365	1, 6	10.78	0.0167

Random effect	Estimate	Standard error (SE)	*Z*	*P*
Donor mouse ID	0.180	0.216	0.83	0.2024

The proportion of CD4^+^IFN-γ^+^ cells was included in the model as a covariate. We report the parameter estimate with the 95% confidence intervals, degrees of freedom (*df*), *F*, and *P* values. The identity of the donor mice used for the in vitro tests was included as a random effect, and we report the parameter estimate with the standard error, the *Z*, and the *P* values. *N* = 8 donor mice and 16 observations. Residual variance was fixed to *π*^2^/3

**Fig. 4:**
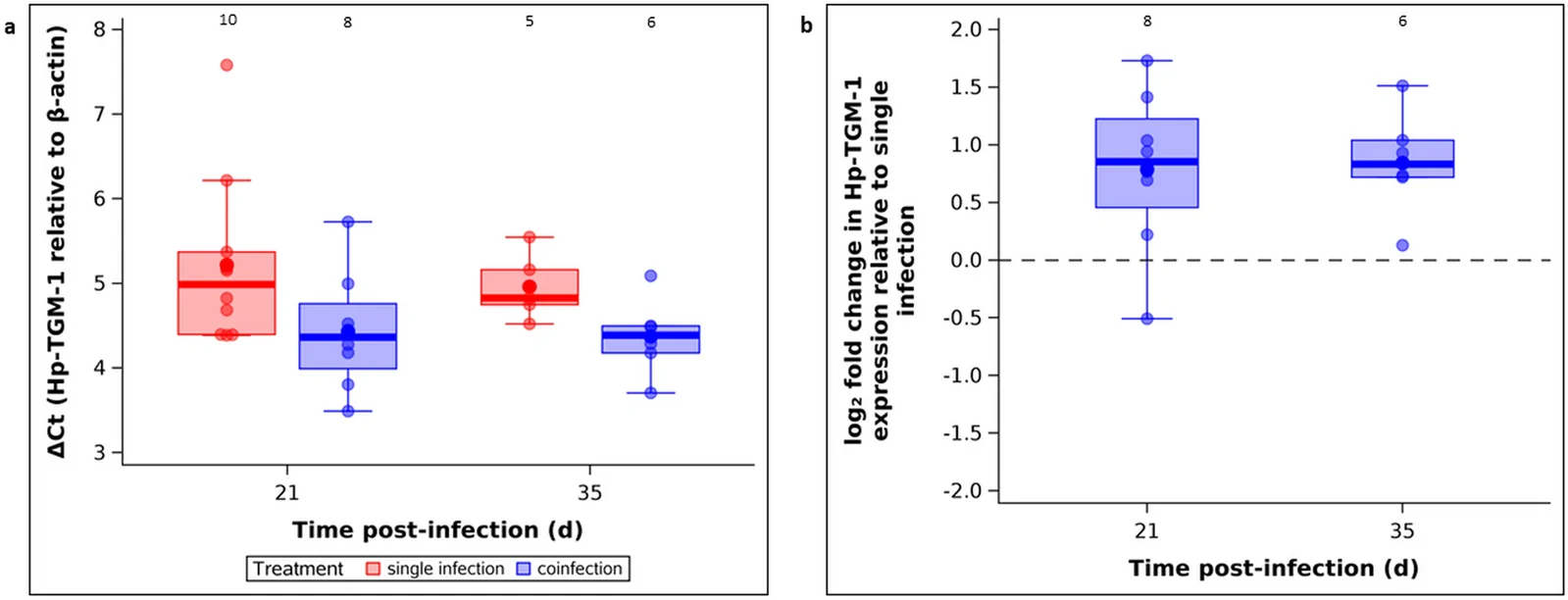
Gene expression of the TGF-β mimic *Hp-TGM-1*. **a** ΔCt of *Hp-TGM-1* (with *β-actin* as the reference gene) in worms from single-infected and coinfected mice at days 21 and 35 post-infection. Worms from coinfected hosts had an upregulated expression (lower ΔCt values) of the *Hp-TGM-1* gene over the two sampling dates (Bonferroni adjusted *P* = 0.0161), the interaction between treatment and time p.i. being nonsignificant (*P* = 0.7322). **b** Log_2_ fold change in *Hp-TGM-1* expression relative to single infected hosts at days 21 and 35 post-infection. Dots represent individual observations, boxes represent the interquartile range (IQR), the horizontal line within each box indicates the median, the larger dot indicates the mean, whiskers extend to the most extreme values within 1.5 × IQR from the quartiles, and points beyond the whiskers indicate outliers. Numbers above the boxes indicate the sample size. The dotted line represents equal *Hp-TGM-1* gene expression in worms from single-infected and coinfected hosts

Finally, we compared the gene expression of the TGF-β mimic *Hp-TGM-1* in worms from single and coinfected hosts, at day 21 and 35 post-Hp infection. To this purpose, we ran a two-way ANOVA. We found that the expression of the *Hp-TGM-1* gene was upregulated in worms from coinfected mice (Table 3; Fig. 4a). However, the magnitude of this upregulation was moderate, as shown by the log_2_ fold change (Fig. 4b).

**Table 3:** Two-way ANOVA investigating the changes in gene expression (ΔCt with *β-actin* as reference gene) of the TGF-β mimic *Hp-TGM-1* in worms from single-Hp infected and coinfected hosts, at day 21 and day 35 post-infection

Effects	*df*	*F*	*P*
Treatment	1, 25	5.64	0.0256
Time p.i.	1, 25	0.30	0.5918
Treatment × time p.i.	1, 25	0.12	0.7322

We report the degrees of freedom (*df*), *F*, and *P* values. *N* = 29

### Experiment 2: effect of experimental evolution in single infected or coinfected hosts on the investment into immunomodulation

**Fig. 5:**
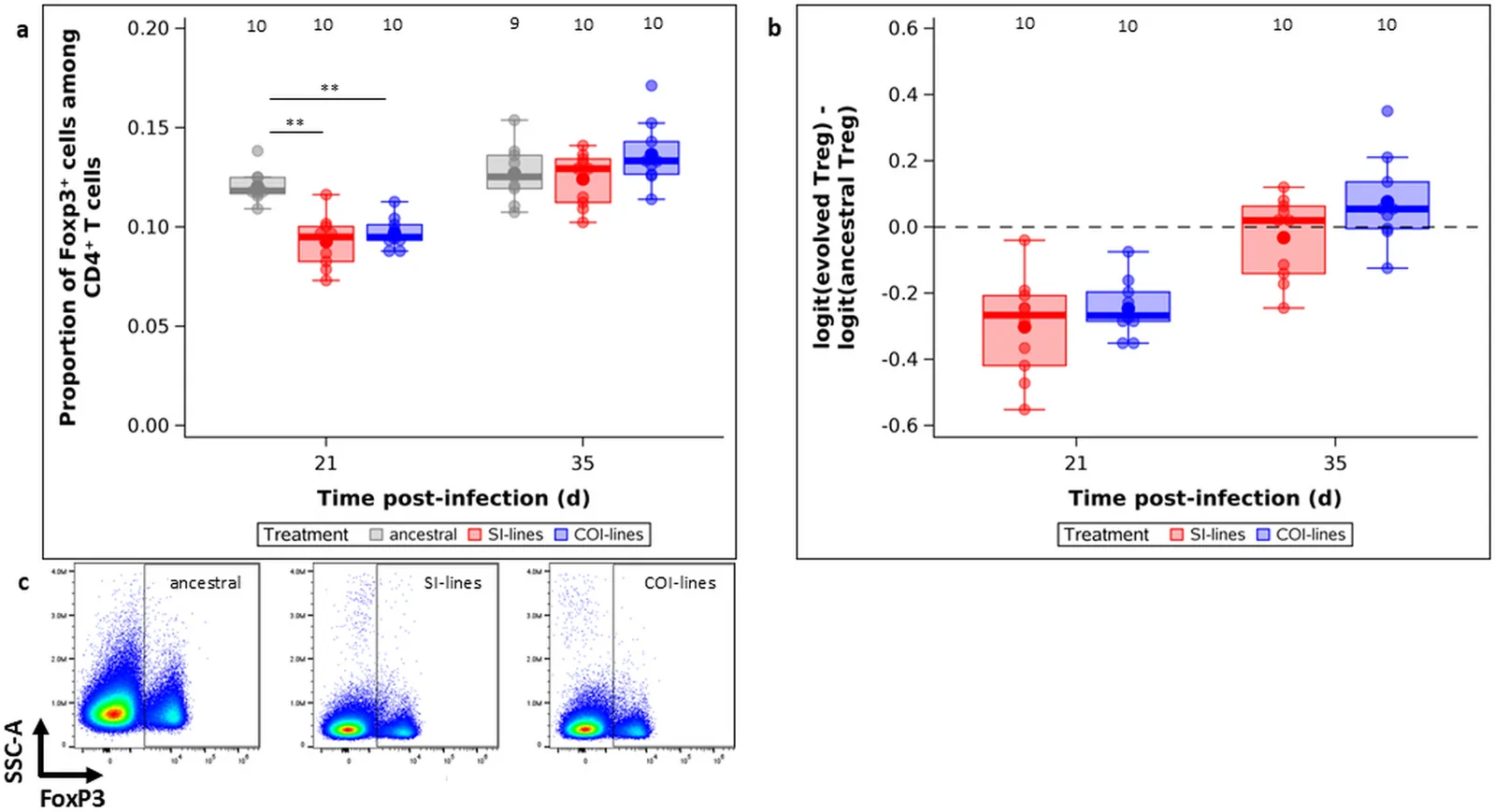
Expansion of Treg cells in vivo. **a** Proportion of Foxp3^+^ cells among CD4^+^ T lymphocytes in the spleen of mice infected with Hp from the ancestral population, from SI-lines, or COI-lines. At day 21 p.i., the proportion of Treg cells was lower in mice infected with Hp from both evolved lines (SI-lines and COI-lines) compared with mice infected with the ancestral population (Bonferroni adjusted *P* < 0.0001 and *P* = 0.0003, respectively), but there was no difference between SI-lines and COI-lines (Bonferroni adjusted *P* = 1). None of the differences between groups was significant at day 35 p.i. (all Bonferroni adjusted *P* values > 0.4). **b** Logit-ratio of the proportion of Foxp3^+^ cells among CD4^+^ T lymphocytes for mice infected with the evolved Hp lines (SI-lines, COI-lines). Logit-ratios are computed as logit(Treg_evolved_) – logit(mean_ancestral_). Over the two sampling dates, there was no difference in the logit-ratio between mice infected with the SI-lines and the COI-lines (Bonferroni adjusted *P* = 0.197), the interaction between treatment and time being nonsignificant (*P* = 0.4703). Dots represent individual observations, boxes represent the interquartile range (IQR), the horizontal line within each box indicates the median, the larger dot indicates the mean, whiskers extend to the most extreme values within 1.5 × IQR from the quartiles, and points beyond the whiskers indicate outliers. Numbers above the boxes indicate the sample size. The dotted line represents equal Treg proportions in mice infected with the ancestral population and mice infected with the evolved lines. **c** Representative biaxial flow cytometry plots (one per experimental group) displaying intracellular Foxp3 versus side scatter area (SSC-A) in CD4^+^ T cells (see Fig. S1 for gating strategy)

We first ran a generalized linear model with a beta-distribution of errors where we compared the proportion of Tregs in mice infected with the ancestral population of Hp, Hp from the SI-lines, or Hp from the COI-lines. We found that the proportion of Tregs strongly differed between mice infected with the ancestral population and the evolved lines at day 21 p.i., and that the difference vanished at day 35 p.i. (Fig. 5a, c), resulting in a highly significant interaction between treatment and time p.i. (Table 4). Post hoc comparisons of least-squares means showed that SI-lines and COI-lines induced a weaker expansion of Tregs compared with the ancestral population at day 21 p.i., but there was no difference between SI-lines and COI-lines, while none of the differences between groups was significant at day 35 post-Hp infection (see Fig. 5 for the post hoc pairwise comparisons). Since this model could not include the replicated Hp line ID, we also ran a linear mixed model where the dependent variable was the logit-ratio between the Tregs of mice infected with SI-lines or COI-lines and the mean Tregs of mice infected with the ancestral Hp population. The results of this model, which included the replicated Hp line ID as a random effect, confirmed the previous finding that there was no difference between the proportion of Tregs in mice infected with the SI-lines and the COI-lines (Fig. 5b; Table 5).

**Table 4:** Generalized linear model with a beta-distribution of errors investigating the effect of Hp serial passage on the population of Treg cells in vivo

Fixed effects		Estimate	95% CI	*df*	*F*	*P*
Treatment				2, 53	10.10	0.0002
Ancestral		0				
SI-lines		−0.299	−0.404/−0.194			
COI-lines		−0.244	−0.348/−0.140			
Time p.i.				1, 53	75.90	< 0.0001
	21	0				
	35	0.057	−0.043/0.158			
Treatment × time p.i.				2, 53	11.98	< 0.0001
SI-lines	21	0				
SI-lines	35	0.273	0.127/0.418			
COI-lines	21	0				
COI-lines	35	0.325	0.182/0.468			

We compared mice infected with the ancestral Hp population and mice infected with the lines of Hp passaged in single-infected (SI-lines) or in coinfected (COI-lines) hosts for eight generations, at days 21 and 35 post-infection. We report the parameter estimate with the 95% confidence interval (95% CI), degrees of freedom (*df*), *F* values, and *P* values. *N* = 59

**Table 5:** Linear mixed model investigating the effect of Hp serial passage on the logit-ratio of Treg cells in vivo

Fixed effects		Estimate	95% CI	*df*	*F*	*P*
Treatment				1, 30	1.85	0.1843
SI-lines		0				
COI-lines		0.048	−0.083/0.179			
Time p.i.				1, 30	67.23	< 0.0001
	21	0				
	35	0.270	0.156/0.385			
Treatment x time p.i.				1, 30	0.53	0.4703
SI-lines	21	0				
SI-lines	35	0				
COI-lines	21	0				
COI-lines	35	0.053	−0.095/0.201			

Random effect	Estimate	SE	*Z*	*P*
Replicated line ID	0.003	0.004	0.81	0.2098
Residual	0.011	0.003	3.73	< 0.0001

We compared mice infected with the lines of Hp passaged in single infected (SI-lines) or in coinfected (COI-lines) hosts for eight generations, at day 21 and 35 post-infection. The identity of the replicated Hp lines was included as a random factor in the model. We report the parameter estimate with the 95% confidence interval (95% CI), degrees of freedom (*df*), and *F* and *P* values. For the random effect, we report the parameter estimate with the standard error (SE) and the *Z* and *P* values. *N* = 8 replicated Hp lines and 40 observations

**Fig. 6:**
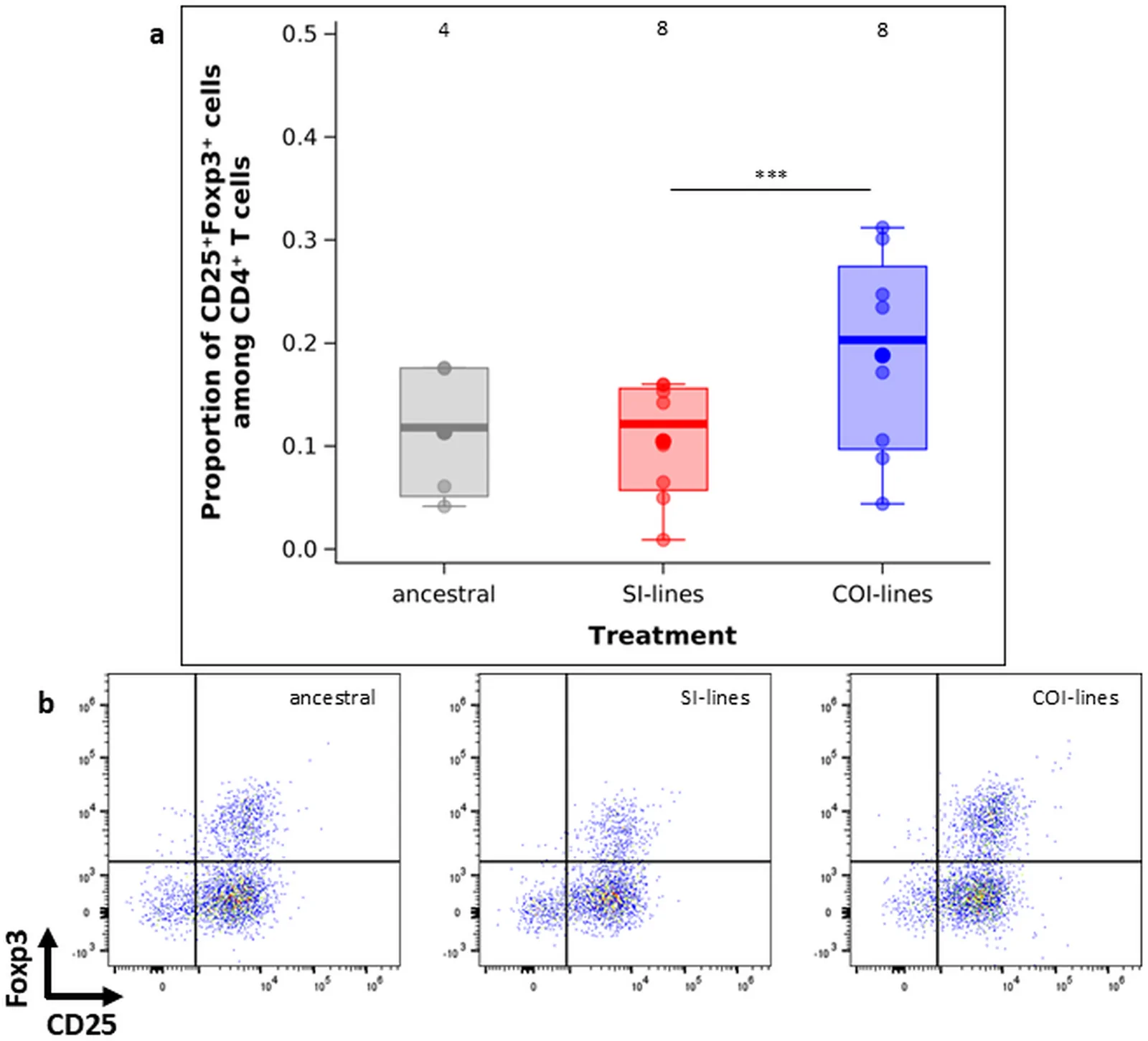
Proportion of CD25^+^Foxp3^+^ cells after in vitro exposure of naïve CD4^+^ T lymphocytes to HES collected from worms of the ancestral population, SI-lines, or COI-lines. **a** The proportion of CD25^+^Foxp3^+^ cells was higher when naïve CD4^+^ T lymphocytes were stimulated with HES collected from COI-lines compared with the SI-lines (Bonferroni adjusted *P* < 0.0001), but the differences were not significant between COI-lines and the ancestral population (*P* = 0.0622), nor between SI-lines and the ancestral population (*P* = 0.3029). Dots represent individual observations, boxes represent the interquartile range (IQR), the horizontal line within each box indicates the median, the larger dot indicates the mean, whiskers extend to the most extreme values within 1.5 × IQR from the quartiles, and points beyond the whiskers indicate outliers. Numbers above the boxes indicate the sample size. **b** Representative biaxial flow cytometry plots (one per experimental group) displaying intracellular Foxp3 versus surface CD25 expression within the CD4^+^ T-cell population (see Fig. S2 for gating strategy)

Then, we investigated whether the capacity of HES to induce an in vitro expansion of Treg and GATA-3^+^ cells differed between the evolved lines and the ancestral population. HES collected from worms in the COI-lines induced a higher expansion of the population of Treg cells in vitro compared with HES collected from worms of the SI-lines (Table 6; Fig. 6), whereas, again, there was virtually no differentiation into GATA-3^+^ cells (Table S6; Fig. S5).

**Table 6:** Generalized linear mixed model with a beta-distribution of errors investigating the effect of Hp serial passage on the capacity of HES to expand the population of Treg cells in vitro

Fixed effects	Estimate	95% CI	*df*	*F*	*P*
Treatment			2, 13	20.99	< 0.0001
Ancestral	0				
SI-lines	−0.332	−0.739/0.074			
COI-lines	0.439	0.079/0.799			
Proportion of CD4^+^IFN-γ^+^ cells	−24.934	−47.056/−2.813	1,13	5.93	0.0300

Random effect	Estimate	SE	*Z*	*P*
Donor mouse ID	0.440	0.377	1.17	0.1217

We compared HES collected from worms of the ancestral population, from worms passaged in single-infected (SI-lines), or from coinfected (COI-lines) hosts for eight generations. The proportion of CD4^+^IFN-γ^+^ cells was included in the model as a covariate. We report the parameter estimate with the 95% confidence interval (95% CI), degrees of freedom (*df*), and *F* and *P* values. The identity of the donor mice used for the in vitro tests was included as a random effect. For the random effect, we report the parameter estimate with the standard error and the *Z* and *P* values. *N* = 4 donor mice and 20 observations. Residual variance was fixed to *π*^2^/3

**Fig. 7:**
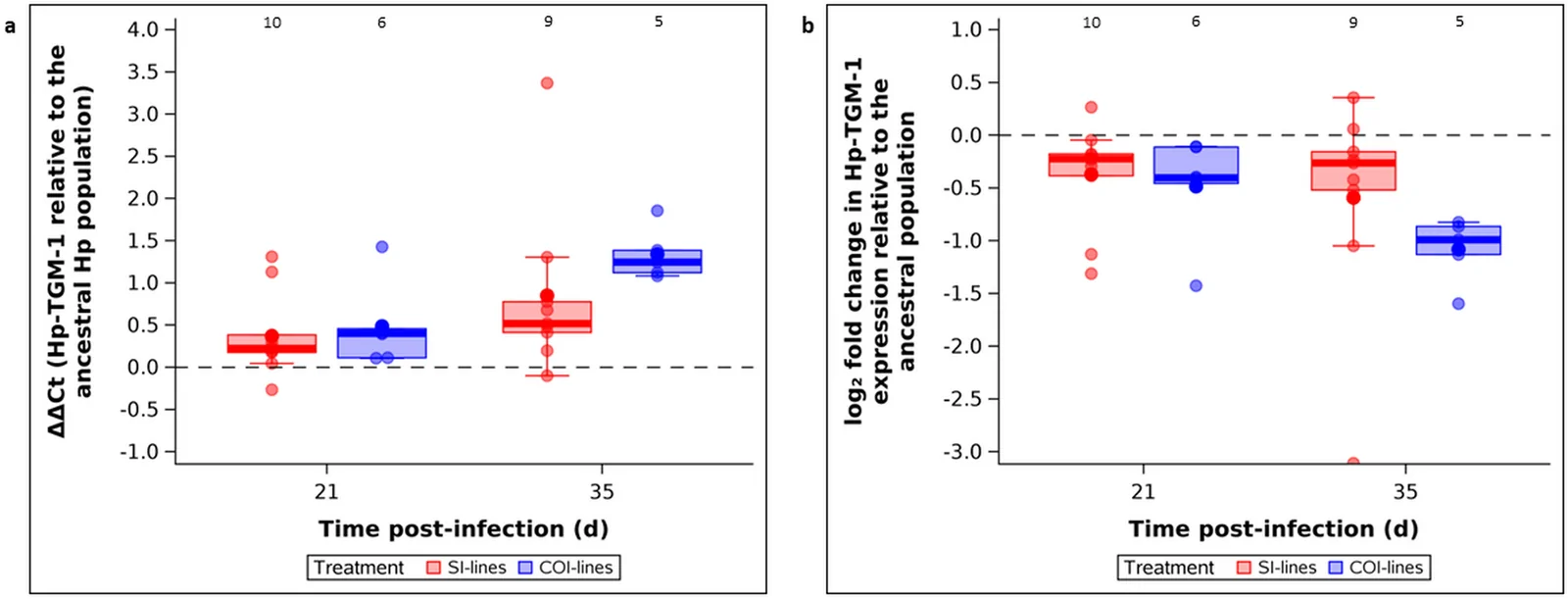
Gene expression of the TGF-β mimic *Hp-TGM-1*. **a** ΔΔCt of *Hp-TGM-1* of worms from the SI-lines or COI-lines, relative to the ancestral population, at days 21 and 35 post-infection. Over the two sampling dates, there was no difference in the *Hp-TGM-1* gene expression in worms from SI-lines and COI-lines (Bonferroni adjusted *P* = 0.3798), the interaction between treatment and time p.i. being nonsignificant (*P* = 0.4037). **b** Log_2_ fold change in *Hp-TGM-1* expression in worms from SI-lines or COI-lines, relative to the ancestral population, at days 21 and 35 post-infection. Dots represent individual observations, boxes represent the interquartile range (IQR), the horizontal line within each box indicates the median, the larger dot indicates the mean, whiskers extend to the most extreme values within 1.5 × IQR from the quartiles, and points beyond the whiskers indicate outliers. Numbers above the boxes indicate the sample size. The dotted line represents equal *Hp-TGM-1* gene expression in worms from the ancestral population and worms from the evolved lines

Finally, we investigated whether the gene expression of the TGF-β mimic *Hp-TGM-1* changed according to the treatment that worms experienced during the passages. To this purpose, we computed the ΔΔCt values relative to the ancestral population. We first ran an intercept-only linear mixed model to investigate whether ΔΔCt values were significantly different from zero, which would indicate a differential gene expression between the ancestral population and the evolved lines. We found that, overall, *Hp-TGM-1* was downregulated in evolved lines compared with the ancestral population [intercept (95% CI) = 0.942 (0.562/1.322), *n* = 8 replicated Hp lines, and 30 observations] (Fig. 7a), but the magnitude of the effect was rather weak, as shown by the log_2_ fold change (Fig. 7b). While being downregulated in the evolved lines, the expression of the *Hp-TGM-1* gene did not differ between SI-lines and COI-lines (Table 7; Fig. 7).

**Table 7:** Linear mixed model investigating the effect of Hp serial passage on the gene expression of the TGF-β mimic *Hp-TGM-1*, at days 21 and 35 post-infection

Fixed effects		Estimate	95% CI	*df*	*F*	*P*
Treatment				1, 20	0.56	0.4625
SI-lines		0				
COI-lines		0.050	−0.793/0.894			
Time p.i.				1, 20	11.35	0.0031
	21	0				
	35	0.531	−0.090/1.152			
Treatment × time p.i.				1, 20	0.73	0.4037
SI-lines	21	0				
SI-lines	35	0				
COI-lines	21	0				
COI-lines	35	0.360	−0.520/1.240			

Random effect	Estimate	SE	*Z*	*P*
Replicated line ID	0.096	0.133	0.72	0.2360
Residual	0.2575	0.088	2.92	0.0017

We compared the ΔΔCt of the lines of Hp passaged in single-infected (SI-lines) or in coinfected hosts (COI-lines) for eight generations, at days 21 and 35 post-infection. The identity of the replicated Hp lines was included as a random effect in the model. We report the estimate with the 95% confidence intervals (95% CI), the degrees of freedom (*df*), and *F* and *P* values. For the random effect, we report the parameter estimate with the standard error and the *Z* and *P* values. *N* = 8 replicated Hp lines and 30 observations

### Experiment 3: effect of experimental evolution in single infected or coinfected hosts on within-host persistence and cumulative egg excretion

**Fig. 8:**
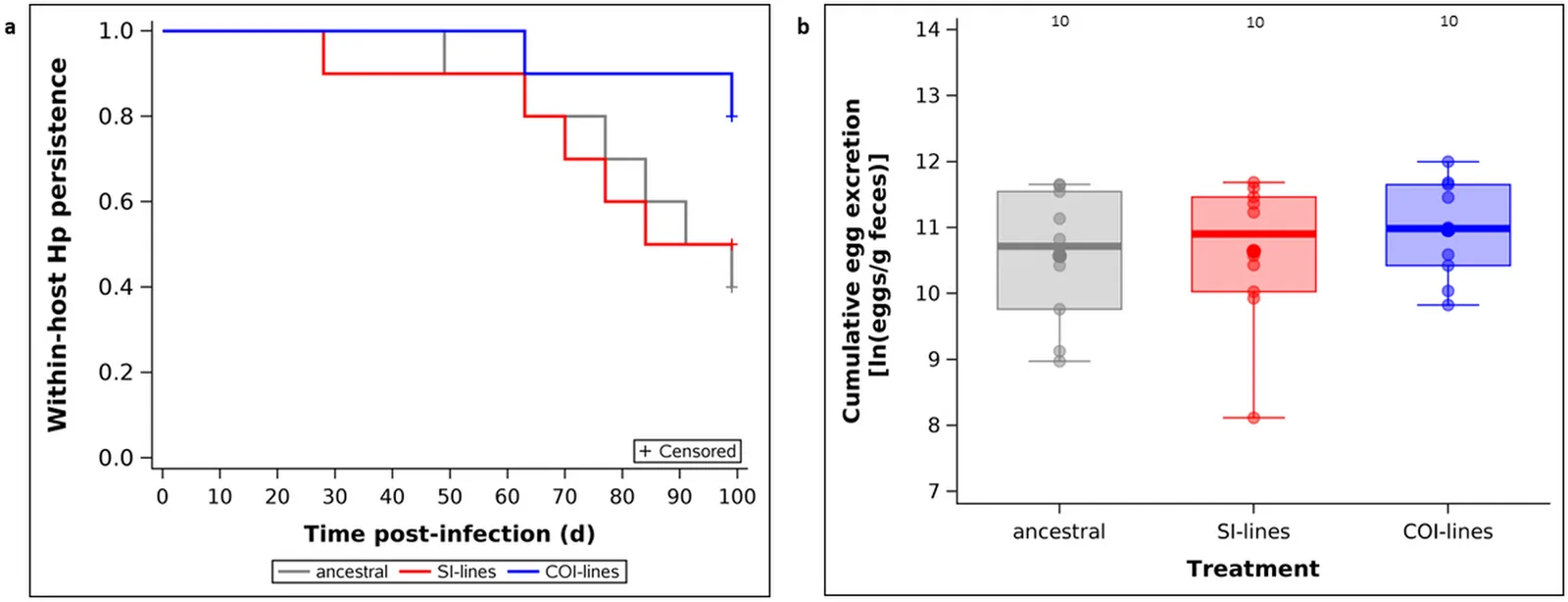
Within-host persistence and cumulative egg excretion. **a** Probability of Hp within-host persistence in the ancestral population and the evolved lines (SI-lines, COI-lines). Crosses indicate censored values. Within-host persistence did not differ between groups (all Sidak adjusted *P* values > 0.2, *n* = 10 mice per group). **b** Cumulative egg excretion [ln-transformed sum of 14 egg excretion values (per g of feces)] in mice infected with worms from the ancestral population and the evolved lines (SI-lines, COI-lines). Cumulative egg excretion did not differ between groups (all Bonferroni adjusted *P* values = 1). Dots represent individual observations, boxes represent the interquartile range (IQR), the horizontal line within each box indicates the median, the larger dot indicates the mean, whiskers extend to the most extreme values within 1.5 × IQR from the quartiles, and points beyond the whiskers indicate outliers. Numbers above the boxes indicate the sample size

We used two metrics (within-host persistence and cumulative egg excretion) to assess the possible demographic consequences of the experimental evolution in single-infected or coinfected hosts. Within-host persistence did not differ between the ancestral Hp population and the evolved lines, nor between the SI-lines and the COI-lines (log-rank test: *χ*^2^_2_ = 3.266, *P* = 0.195, *n* = 10 per treatment; all Sidak adjusted *P*-values of pairwise comparisons > 0.2) (Fig. 8a). Similarly, the cumulative egg excretion up to day 99 p.i. did not differ between the ancestral Hp population and the evolved lines (one-way ANOVA: *F*_2, 27_ = 0.48, *P* = 0.6235) (Fig. 8b).

## Discussion

We predicted that Hp should adjust its investment into the production of immunomodulatory effectors, according to the infectious status of the host. More specifically, we predicted that, when Hp infects hosts that already harbor a microparasite infection, it should reduce its investment into immunomodulation. This prediction was based on a relatively straightforward argument: if immunomodulation favors microparasite multiplication the host can be overwhelmed, and if the host dies, the fitness of the immunosuppressive parasite can be jeopardized. We ran two experiments to test this hypothesis, a within-generation and an experimental evolution, and we did not find evidence in support of it. Although coinfected mice had a lower proportion of Treg cells compared with single infected mice, this difference could not be reproduced when focusing on two mechanisms solely under Hp control. HES collected from worms in single infected and coinfected hosts had similar capacity to differentiate naïve CD4^+^ T lymphocytes into Treg cells in vitro, and the gene expression of the TGF-β mimic *Hp-TGM-1* was upregulated in worms from the coinfection group, in contrast to the predicted sign of the effect. Therefore, these results suggest that the reduced Treg response in coinfected mice is driven by host factors rather than by Hp factors.

The capacity of helminths to interfere with the host immune response has attracted considerable attention in recent years for several reasons, including the possibility to harness these immunomodulatory properties to treat inflammatory and immune-mediated diseases [52–59]. However, only a few clinical trials have been conducted, with mixed results, questioning the possibility of extending the results obtained in animal models to human diseases [77–79]. One factor that has been overlooked as a potential underlying reason for these mixed results is the extent to which helminths have fixed or plastic immunomodulatory strategies. Under a fixed strategy, parasites are expected to have similar investments in immunomodulatory effectors, whatever the environment provided by the host. However, such a strategy might not be the most effective one for several reasons. First, although homeothermic hosts are supposed to provide a relatively stable environment, for instance, in terms of temperature or resources, they can differ in their capacity to mount an effective immune response [80, 81]. If producing immunomodulatory effectors entails an energetic cost, parasites infecting hosts that do not provide a harmful environment should not waste resources to produce unneeded excretory/secretory products. Another very important aspect likely to alter the immune environment is whether the host already harbors other parasites/pathogens at the time of helminth infection [12]. Coinfection has indeed the potential to polarize the immune response towards Th1 or Th2 effectors depending on the nature and order of coinfecting parasites [18, 19, 27, 30, 82–87]. More interestingly, coinfection makes the immunomodulatory strategy particularly risky, if immune interference enhances the probability of host death due to the concomitant infection. This is particular true when the coinfecting organism is a microparasite with a fast replicating rate and the capacity to rapidly overwhelm host defenses. Therefore, again, expressing a fixed strategy of immunomodulation whatever the environmental conditions encountered by nematodes is likely to be too risky.

In agreement with this general prediction, we found that coinfection induced a lower expansion of the population of Treg cells compared with single-infected mice. Previous work has consistently shown that infection with Hp induces a host response characterized by an expansion of the population of Treg cells that can provide protection against immune-mediated damage [49–51]. The comparison between control noninfected and single-Hp infected hosts confirmed that Hp induced an expansion of the population of Treg cells. However, as mentioned above, coinfected mice did not show an increased proportion of Treg cells, as the proportion among CD4^+^ T lymphocytes did not differ between noninfected and coinfected mice. Although this result could be interpreted as evidence for a strategic adjustment of Hp investment into immunomodulatory effectors, it is important to note that the in vivo response is influenced by both host and parasite factors. Therefore, the reduced expansion of Treg cells in coinfected hosts may reflect a host-driven response rather than a direct effect of Hp-induced immunomodulation.

Therefore, to disentangle the host response from any Hp manipulation, we directly assessed two mechanisms that are solely under Hp control: the capacity of HES to induce an expansion of Tregs in vitro and the gene expression of the TGF-β mimic *Hp-TGM-1*. Hp excretes/secretes hundreds of biomolecules that have, among other functions, the capacity to interfere with the host immune response and induce the expansion of the population of Treg cells [45, 61–65, 67]. In agreement with these previous results, we showed that when exposed to heat-inactivated HES or PBS, naïve CD4^+^ lymphocytes did not differentiate into CD25^+^Foxp3^+^ cells, while exposure to HES collected from worms in single-infected hosts induced an expansion of the population of Treg cells. Therefore, as expected, HES did induce an anti-inflammatory response by inducing a Treg differentiation. Given that we found a reduced expansion of the Treg population in coinfected mice in vivo, if this result was due to an adjustment in the production of HES of worms in coinfected hosts, we should have found a lower capacity of HES collected from worms in coinfected hosts to differentiate naïve CD4^+^ lymphocytes into Treg cells. However, we did not find any difference between HES collected from worms in single and coinfected hosts.

As mentioned above, Hp excretes/secretes hundreds of proteins. Among them, one group of proteins has received particular attention: Hp-TGM. The Hp genome has a family of genes that produce proteins structurally and functionally similar to the mammalian anti-inflammatory cytokine TGF-β. TGF-β is one of the cytokines that induces the differentiation of CD4^+^ lymphocytes into regulatory T cells [88]. Therefore, given that coinfected mice had a lower proportion of Treg cells, we expected that worms from coinfected hosts should have a downregulated expression of the *Hp-TGM-1* gene. Contrary to this prediction, we found that worms in coinfected hosts upregulated the expression of *Hp-TGM-1* (although the magnitude of the effect was rather modest). Thus, this result indicates that there was no association between the in vivo Treg response and the expression of the TGF-β mimic that is solely under parasite control. Therefore, globally, we did not find evidence supporting the hypothesis that the in vivo results might be explained by the Hp adjustment of investment into immunomodulatory effectors. The impaired Treg response in coinfected mice could thus be solely under host control, and could reflect the host need to effectively control the high replication rate of Py.

After having investigated the within-generation effects, we wished to go a step further in the study of the consequences of coinfection on the Hp-induced immunomodulation. To this purpose, we also adopted an experimental evolution approach that allowed us to investigate the between-generation changes in immunomodulation when Hp experienced either a single infected or a coinfected host. On the basis of the same rationale mentioned above, we predicted that Hp lines maintained in coinfected hosts should evolve toward lower levels of immunomodulation. After eight generations of exposure to the environment provided by single infected or coinfected hosts, mice infected with the evolved lines had a weaker expansion of Tregs in vivo compared with mice infected with the ancestral population, but there was no difference between the SI-lines and the COI-lines, showing that hosts similarly responded to the two evolved lines. It should be reminded that mice were infected with the evolved lines during single infection trials (as it was for the ancestral population), isolating the effect of the selection regime from any possible confounding effect of the coinfection. Therefore, this result shows that there was no statistically significant difference in host response between lines evolving in coinfected hosts and lines dwelling in single infected hosts. This finding was mirrored by the analysis of the *Hp-TGM-1* gene expression, showing that evolved lines downregulated it compared with the ancestral population (although the magnitude of the effect was rather small), with no difference between SI-lines and COI-lines. The only result suggesting a possible divergent microevolutionary response between SI-lines and COI-lines concerned the capacity of HES to induce a Treg differentiation in vitro, since we found that HES collected from worms of the COI-lines induced a stronger Treg differentiation compared with HES collected from worms of the SI-lines. However, the direction of the difference did not fit the prediction, since we expected worms in the COI-lines to reduce their investment into the immunomodulatory effectors. Therefore, overall, there was no evidence for a microevolutionary adjustment of the investment into immunomodulation. However, it is possible that eight generations of selection were insufficient to observe a microevolutionary response. The rate of evolutionary change depends on both the strength of selection and the amount of additive genetic variation present. Since the Hp population used in this study has been maintained in the laboratory for many generations, it is possible that genetic variation has been reduced under laboratory conditions, limiting the potential for evolutionary change (see ref. [89]).

After eight passages in single infected or in coinfected hosts, Hp lines had similar within-host persistence time and similar cumulative egg excretion compared with the ancestral population. Although these traits cannot be considered as proxies of individual fitness since they refer to the whole infra-host population of worms, they nevertheless provide useful insights on the possible epidemiological consequences of being consistently exposed to single infected or coinfected hosts. As for the investment into immunomodulatory molecules, we did not find any difference between lines (nor between the ancestral population and the evolved lines) suggesting that Hp lines maintained in coinfected hosts performed similarly well than lines maintained in single infected mice. It should be reminded, however, that the assessment of within-host persistence and cumulative egg excretion was only conducted during single infection trials. We cannot therefore exclude that COI-lines might have fared better when tested in coinfection trials.

Overall, these results might contribute to a better understanding of the consequences of coinfection for the epidemiology and the severity of malaria symptoms. Malaria and helminths are prevalent human parasites that occur in the same geographical zones (mostly the intertropical area) and often coinfect the same hosts [90]. The severity of malaria infection is often exacerbated in helminth coinfected humans, although a lot of variation exists depending on multiple factors (species of coinfecting helminth, order of infection, or host age) [27, 91]. Drivers of severity of disease symptoms often involve direct effects due to the proliferation of the pathogen and indirect effects due to the pro-inflammatory response induced by *Plasmodium* and the associated damage [31, 92]. We found that coinfected hosts had a reduced expansion of the population of Treg cells compared with single-infected hosts, and that this reduced Treg expansion is probably under host control. Therefore, despite the lack of adjustment in the investment into immunomodulatory effectors by the nematode, the immune response of coinfected hosts was less regulated than in single-infected mice. While this might be beneficial to the host to control Py multiplication, it might also incur costs in terms of inflammatory and immune-mediated damage. However, we would also like to stress that it is often difficult to extrapolate results obtained under controlled laboratory conditions to predict the epidemiology and evolutionary responses under the diverse conditions encountered under natural environments. We therefore advocate for more work in the field to explore to what extent our results also apply to more natural conditions.

We would like to acknowledge a few limitations of our work. The rationale beyond the prediction that Hp should invest less into immunomodulatory effectors in coinfected hosts is based on the idea that if hosts are overwhelmed by rapidly multiplying microparasites they might succumb, and as such, jeopardize the fitness of helminths establishing chronic infections. However, in our experimental setup, none of the coinfected mice succumbed to the infection. In a previous paper [27], we showed that the severity of the disease is exacerbated in coinfected hosts, but only when the infections occur in a specific order: Hp infects first and Py infects second. Under these circumstances, Py becomes substantially more virulent because it takes advantage of the modifications of the immune response induced by a preceding Hp infection. However, when Py infects hosts that are already infected by Hp, it may be too late for the worm to adjust its immunomodulatory strategy. Conversely, we speculated that when Hp infects hosts that already harbor the Py infection, it may perceive environmental cues due to the co-infection that could prompt a timely readjustment.

Our assessment of immunomodulation was based on the expansion of the population of Treg cells in vivo and in vitro (following the stimulation of naïve CD4^+^ T lymphocytes with HES) and on the gene expression of the TGF-β mimic *Hp-TGM-1*. However, we cannot exclude that other regulatory pathway, such as those involving myeloid-derived suppressor cells (MDSCs) [93], tolerogenic dendritic cells, or alternatively activated macrophages [4, 44], might have been affected by coinfection in the predicted way.

The capacity of HES to stimulate the expansion of the population of Treg cells in vitro was assessed by culturing adult worms over 4 days. Obviously, the environmental conditions experienced in the culture flasks are essentially different from the within-host conditions and it is therefore likely that HES also included molecules specifically produced in response to the culture conditions. Future advances in this area might involve the use of intestinal organoids to culture parasites ex vivo.

We also found that two key epidemiological traits (the within-host persistence and the cumulative egg excretion) did not differ between the ancestral Hp population, SI-lines, and COI-lines when tested during single-infection trials, suggesting that, overall, the conditions experienced in coinfected hosts did not modify the capacity of Hp to persist and shed eggs. However, it should be noted that these traits refer to the whole infra-host population of worms, since it is not possible to assess individual fitness during in vivo infections. This also raises the question of the possible effect of variability between individual worms within hosts and the associated increase in heterogeneity of the response variables.

## Conclusions

These results show that while malaria coinfection produced a change in the expansion of the population of Tregs in vivo, in agreement with our prediction, this does not correspond to a strategic adjustment of the Hp investment into host immunomodulation. The results of the experimental evolution approach corroborated this view, since evolved Hp lines generally induced comparable host responses and had similar within-host persistence and egg excretion. Further work is needed to assess the extent to which these results can be extrapolated in order to inform the epidemiology of helminth–malaria coinfection, and to predict the severity of malaria symptoms in hosts coinfected with gastrointestinal helminths.

## Supplementary Information

Additional file 1.

## Data Availability

All data and codes have been deposited in Zenodo (10.5281/zenodo.18339732).
