# Supplementary material for: In vivo reduction of Treg expansion in rodent helminth–malaria coinfection
Source: Parasit Vectors. 2026 Jun 13;19:342. doi: 10.1186/s13071-026-07465-3 (PMC13491614; doi:10.1186/s13071-026-07465-3)
Supplement: Supplementary file 1 [file 13071_2026_7465_MOESM1_ESM.pdf]

Supplementary Material

In vivo reduction of Treg expansion in rodent helminth-malaria coinfection

Luc Bourbon[1*], Aloïs Dusuel[2*], Nicolas Pernet[3], Mickaël Rialland[2], Benjamin Roche[4], Bruno Faivre[1], Gabriele Sorci[1#]

1. Biogéosciences, CNRS UMR 6282, Université Bourgogne Europe, 6 Boulevard Gabriel, 21000 Dijon, France

2. Center for Translational and Molecular Medicine UMR1231, Université Bourgogne Europe, 21000 Dijon, France; LabEx LipSTIC, 21000 Dijon, France

3. Plateforme ImaFlow – US58 BioSanD, UFR Sciences de Santé, 7 Boulevard Jeanne d'Arc, 21079 Dijon cedex, France

4. MIVEGEC, IRD, CNRS, Université de Montpellier, Montpellier, France

#corresponding author: gabriele.sorci@u-bourgogne.fr

*equal contribution

Supplementary tables and figures

Table S1. Spectral flow cytometry panels.

A) *In vivo* experiment: staining of mouse splenocytes.

Emission (nm)	Target	Fluorophore	Clone	Supplier	Cat. ID
Viability					
473	/	LIVE/DEAD Blue	/	Invitrogen	L23105
Surface markers					
388	CD45	BUV395	30-F11	BD Bioscience	564279
615	CD19	BUV615	1D3	BD Bioscience	751213
661	CD8α	BUV661	53-6.7	BD Bioscience	750023
605	CD3	BV605	17A2	BD Bioscience	564009
700	CD4	AF700	RM4-5	BD Bioscience	557956
Intracellular markers					
774	FOXP3	PE-Cy7	FJK-16S	Invitrogen	25-5773-82

B) *In vitro* experiment: staining of $CD4^+$ T cells.

Emission (nm)	Target	Fluorophore	Clone	Supplier	Cat. ID
Viability					
473	/	LIVE/DEAD Blue	/	Invitrogen	L23105
Surface markers					
421	CD25	BV421	3C7	BioLegend	101923
700	CD4	AF700	RM4-5	BD Biosciences	557956
Intracellular markers					
693	GATA-3	BB700	L50-823	BD Biosciences	566642
711	IFN-γ	BV711	XMG1.2	BD Biosciences	564336
774	FOXP3	PE-Cy7	FJK-16S	Invitrogen	25-5773-82

Table S2. Primer sequences used for the RT-qPCR of *Hp-TGM-1* gene.

**Target**	**Forward sequence (5' to 3')**	**Reverse sequence (5' to 3')**
β-Actin	ATGGAGGGGAATACAGCCC	TTCTTTGCAGCTCCTTCGTT
Hp-TGM-1	CTACGCAACCGGCTCAAAAGT	TAGTGTGCGAATTCCGTCCTT

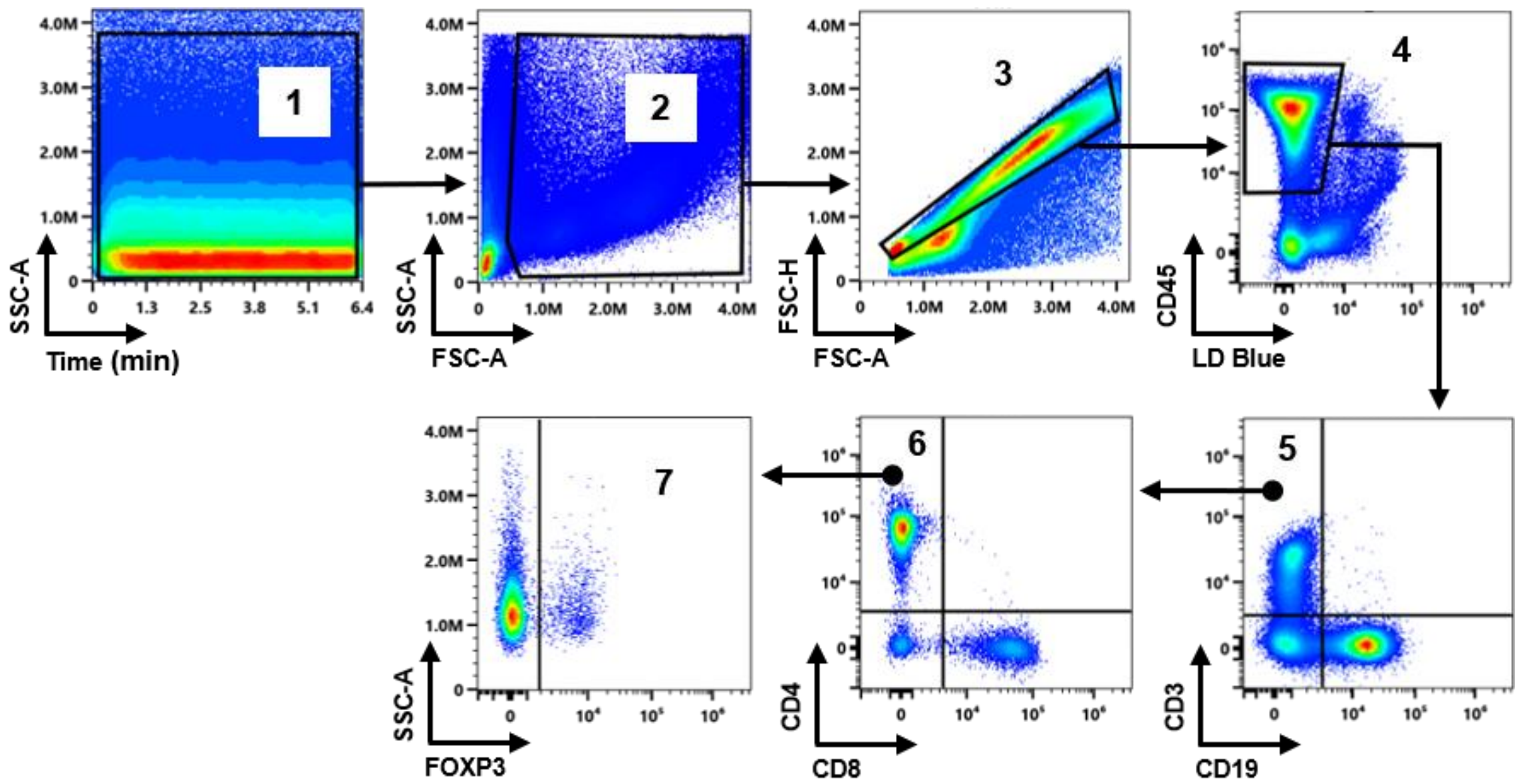

Figure S1. Gating strategy used in flow cytometry analysis to identify Treg cell subset within mouse total splenocytes. (1) Time parameter to exclude microfluidic fluctuations. Selection of (2) splenocytes without debris and (3) single cells. Selection of (4) live LIVE/DEAD-Blue$^-$ and immune CD45$^+$ cells. Selection of (5) CD19$^-$CD3$^+$ T cells. Selection of (6) CD8$^-$CD4$^+$ helper T cells. Selection of (7) FOXP3$^+$ Treg cell subset within CD4$^+$ T lymphocytes.

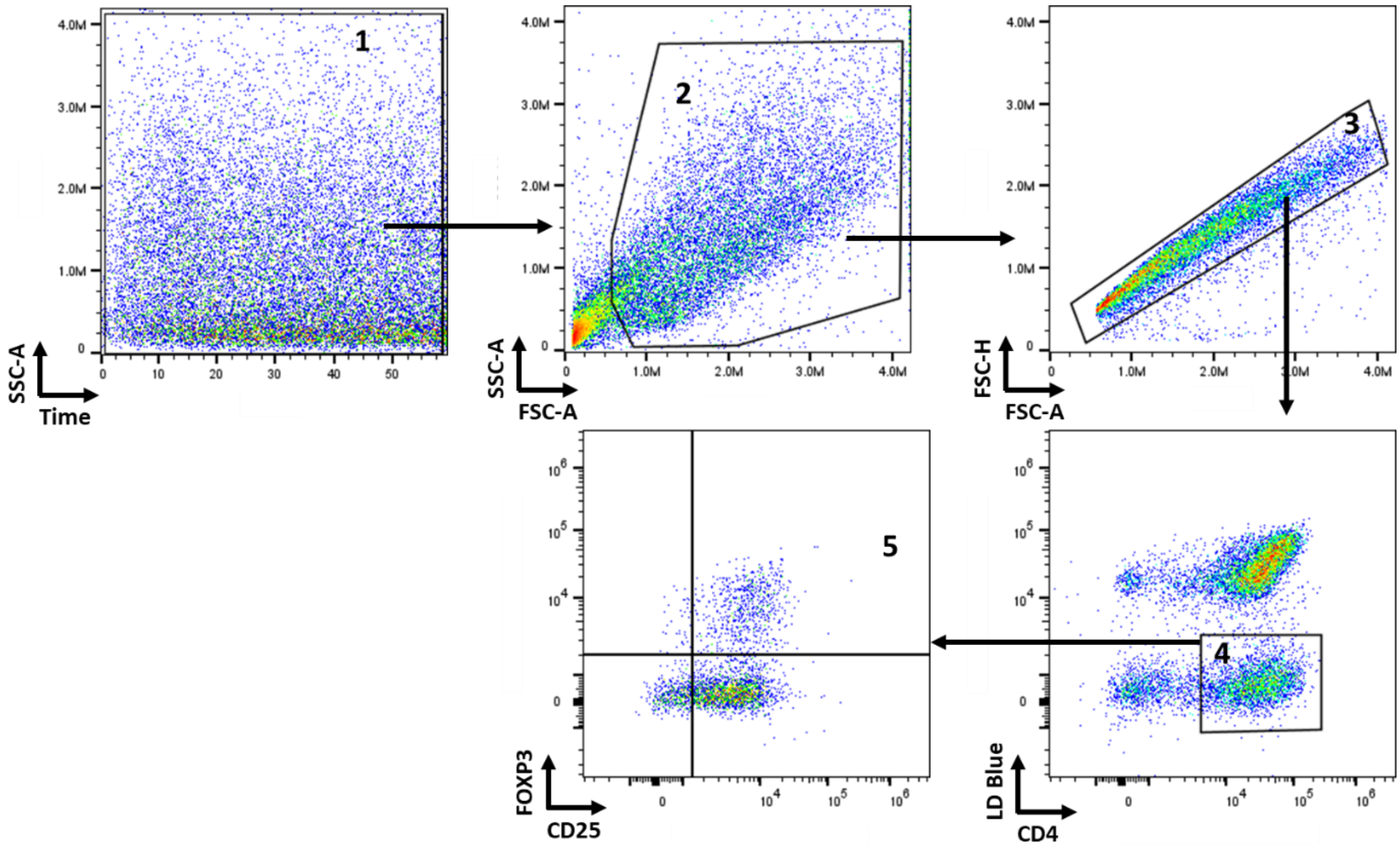

Figure S2. Gating strategy used in flow cytometry analysis to identify Treg cells upon *in vitro* stimulation of naive CD4$^+$ T cells with HES. (1) Time parameter to exclude microfluidic fluctuations. Selection of (2) cells without debris and (3) single cells. Selection of (4) live LIVE/DEAD-Blue$^-$ and CD4$^+$ cells. Selection of (5) CD25$^+$Foxp3$^+$ T cells.

Table S3. Generalized linear mixed model with a beta-distribution of errors exploring the capacity of HES collected from worms in single Hp infected host to differentiate naïve $CD4^+$ T lymphocytes into $CD25^+Foxp3^+$ cells *in vitro* compared to heat-inactivated HES and PBS. The proportion of $CD4^+IFN\text{-}\gamma^+$ cells was included in the model as a covariate. We report the parameter estimate with the 95% confidence intervals, degrees of freedom (df), F and p values. The identity of the donor mice was included in the model as a random effect and we report the parameter estimate with the standard error, the Z and p values. N = 4 donors and 12 observations. Residual variance was fixed to $\pi^2/3$.

**Fixed effects**		**Estimate**	**95% CI**	**df**	**F**	**p**
Treatment				2,5	18.87	0.0047
	single infection HES	0				
	Heat-inactivated HES	-1.951	-3.113/-0.790			
	PBS	-2.321	-3.467/-1.175			
Proportion of $CD4^+IFN\text{-}\gamma^+$ cells		-8.286	-17.116/0.544	1,5	5.82	0.0607
**Random effect**		**Estimate**	**SE**	**Z**	**p**	
Donor mouse id		0.007	0.087	0.08	0.4684	

Table S4. Generalized linear mixed model with a beta-distribution of errors exploring the capacity of HES collected from worms in single Hp infected host to differentiate naïve $CD4^+$ T lymphocytes into GATA-3^+ cells *in vitro* compared to heat-inactivated HES and PBS. The proportion of $CD4^+$IFN-γ^+ cells was included in the model as a covariate. We report the parameter estimate with the 95% confidence intervals, degrees of freedom (df), F and p values. The identity of the donor mice was included in the model as a random effect and we report the parameter estimate with the standard error, the Z and p values. N = 4 donors and 12 observations. Residual variance was fixed to $\pi^2/3$.

**Fixed effects**		**Estimate**	**95% CI**	**df**	**F**	**p**
Treatment				2,5	1.41	0.3276
	single infection HES	0				
	heat-inactivated HES	2.198	-1.185/5.582			
	PBS	1.657	-1.317/4.632			
Proportion of $CD4^+$IFN-γ^+ cells		-2.284	-28.393/23.825	1,5	0.05	0.8310
**Random effect**		**Estimate**	**SE**	**Z**	**p**	
Donor mouse id		1.385	1.593	0.87	0.1924	

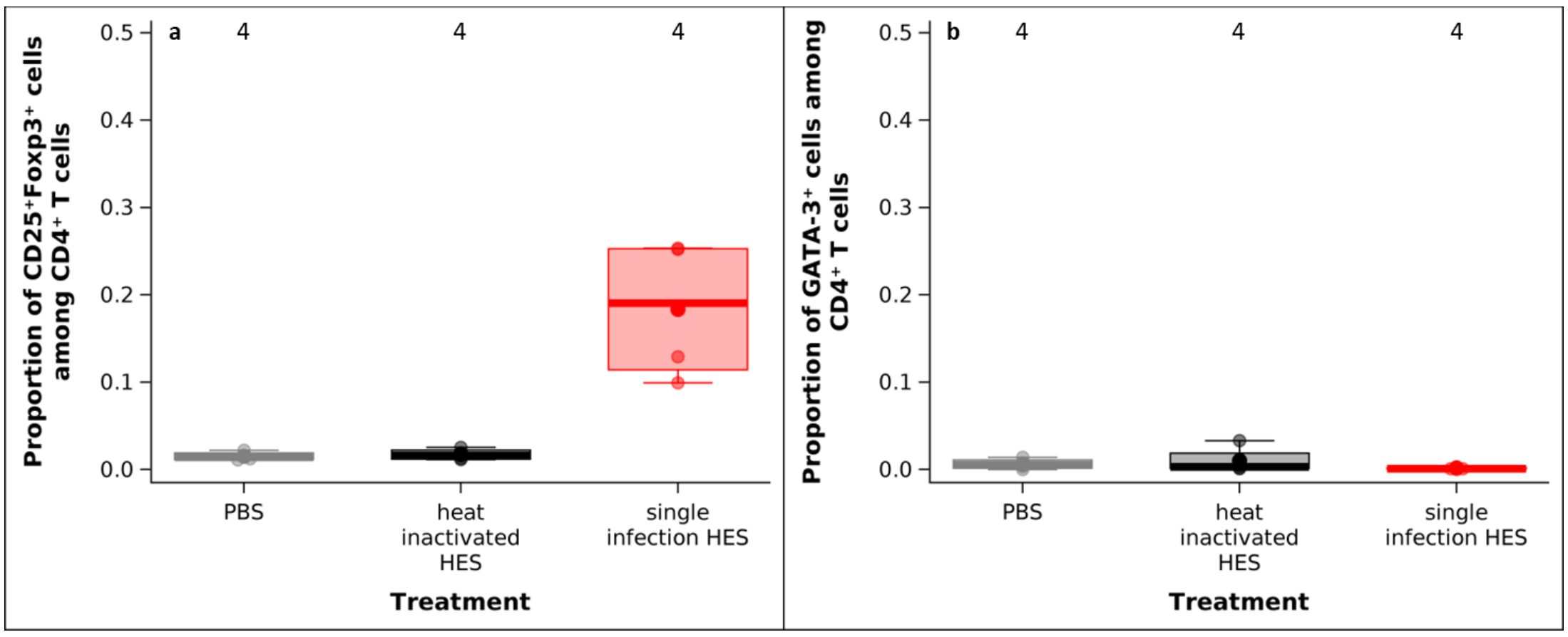

Figure S3. *In vitro* differentiation of naïve CD4$^+$ T lymphocytes into CD25$^+$Foxp3$^+$ and GATA-3$^+$ cells. a) Proportion of CD25$^+$Foxp3$^+$ cells after *in vitro* exposure of naïve CD4$^+$ T lymphocytes to PBS, heat-inactivated HES collected from worms in single infected hosts, or HES collected from worms in single infected mice. The proportion of Treg cells was significantly different between the HES collected from worms in single infected hosts and the PBS and heat-inactivated groups (Bonferroni adjusted $p = 0.008$ and $p = 0.\ 0173$, respectively). b) Proportion of GATA-3$^+$ cells after *in vitro* exposure of naïve CD4$^+$ T lymphocytes to PBS, heat-inactivated HES collected from worms in single infected hosts, or HES collected from worms in single infected mice. The proportion of GATA-3$^+$ cells did not differ between groups (all Bonferroni adjusted p's > 0.3). Dots represent individual observations; boxes represent the interquartile range (IQR); the horizontal line within each box indicates the median; the larger dot indicates the mean; whiskers extend to the most extreme values within 1.5 × IQR from the quartiles; and points beyond the whiskers indicate outliers. Numbers above the boxes indicate the sample size.

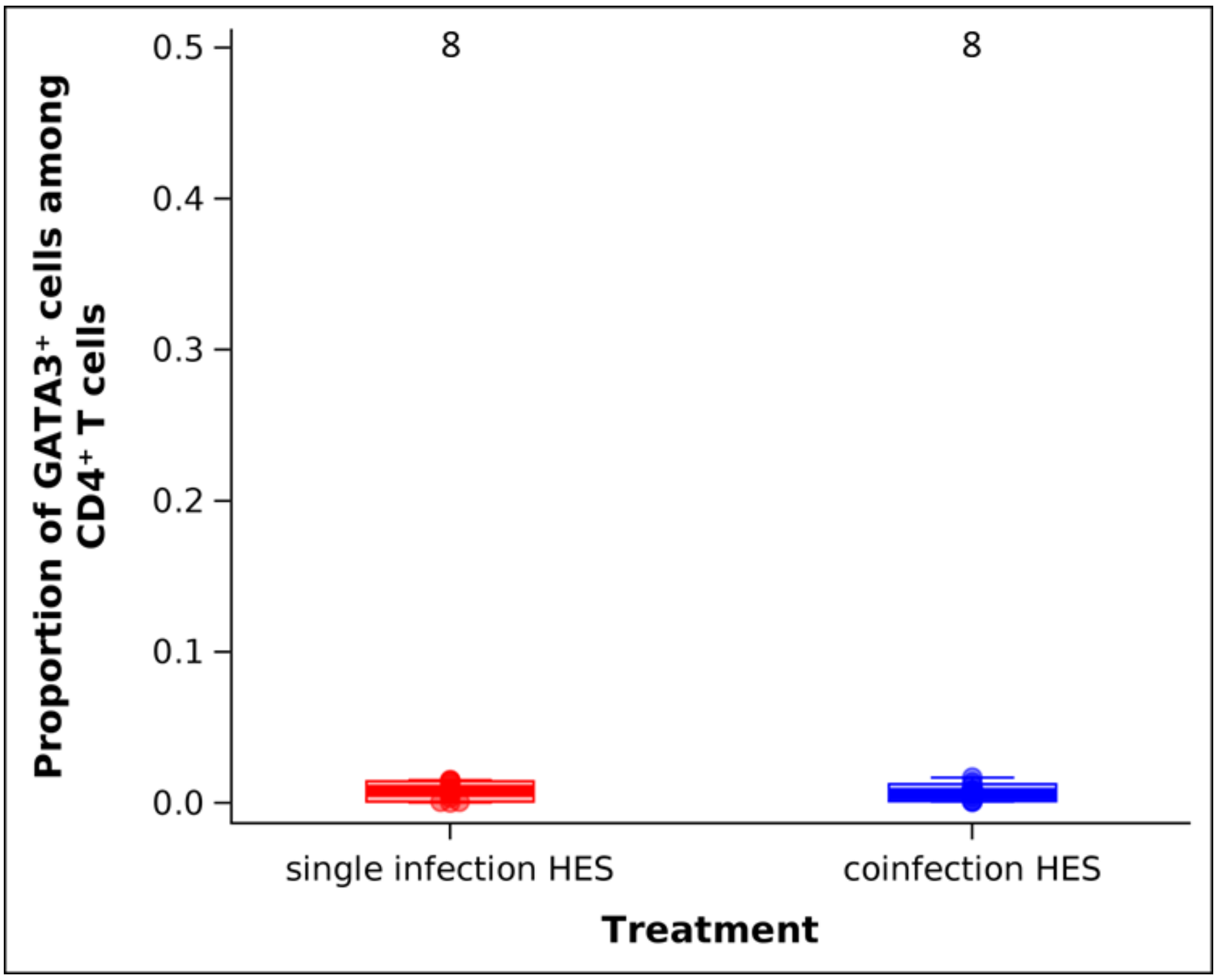

Figure S4. *In vitro* differentiation of naïve CD4$^+$ T lymphocytes into GATA-3$^+$ cells. Proportion of GATA-3$^+$ cells after *in vitro* exposure of naïve CD4$^+$ T lymphocytes to HES collected from worms in single infected mice, or from worms in coinfected mice. The proportion of GATA-3$^+$ cells did not differ between the single infection HES and the coinfection HES (p = 0.5512). Dots represent individual observations; boxes represent the interquartile range (IQR); the horizontal line within each box indicates the median; the larger dot indicates the mean; whiskers extend to the most extreme values within 1.5 × IQR from the quartiles; and points beyond the whiskers indicate outliers. Numbers above the boxes indicate the sample size.

Table S5. Generalized linear mixed model with a beta-distribution of errors exploring the effect of treatment (single Hp infection, coinfection) on the capacity of HES to expand the population of GATA-3$^+$ cells *in vitro*. We report the parameter estimate with the 95% confidence intervals, degrees of freedom (df), F and p values. The identity of the donor mice used for the *in vitro* tests was included as a random effect, and we report the parameter estimate with the standard error, the Z and p values. N = 8 donor mice and 16 observations. Residual variance was fixed to $\pi^2/3$.

**Fixed effect**		**Estimate**	**95% CI**	**df**	**F**	**p**
Treatment				2,14	0.17	0.8429
	ancestral	0				
	SI-lines	-0.020	-0.333/0.294			
	COI-lines	0.048	-0.262/0.358			
**Random effect**		**Estimate**	**SE**	**Z**	**p**	
Donor mouse id		0.053	0.053	1.01	0.1574	

Table S6. Generalized linear mixed model with a beta-distribution of errors investigating the effect of Hp serial passage on the capacity of HES to expand the population of GATA-3^{+} cells *in vitro*. We compared HES collected from worms of the ancestral population, from worms passaged in single infected (SI-lines) or in coinfected (COI-lines) hosts for eight generations. The proportion of CD4^{+}IFN-γ^{+} cells was included in the model as a covariate. We report the parameter estimate with the 95% confidence interval (95% CI), degrees of freedom (df), F and p values. The identity of the donor mice used for the *in vitro* tests was included as a random effect. For the random effect, we report the parameter estimate with the standard error, the Z and p values. N = 4 donor mice and 20 observations. Residual variance was fixed to $\pi^2/3$.

**Fixed effects**		**Estimate**	**95% CI**	**df**	**F**	**p**
Treatment				1,6	0.40	0.5512
	single Hp infection	0				
	coinfection	-0.104	-0.508/0.300			
Proportion of CD4^{+}IFN-γ^{+} cells		4.415	-18.749/27.579	1,6	0.22	0.6574
**Random effect**		**Estimate**	**SE**	**Z**	**p**	
Donor mouse id		1.481	0.942	1.57	0.0579	

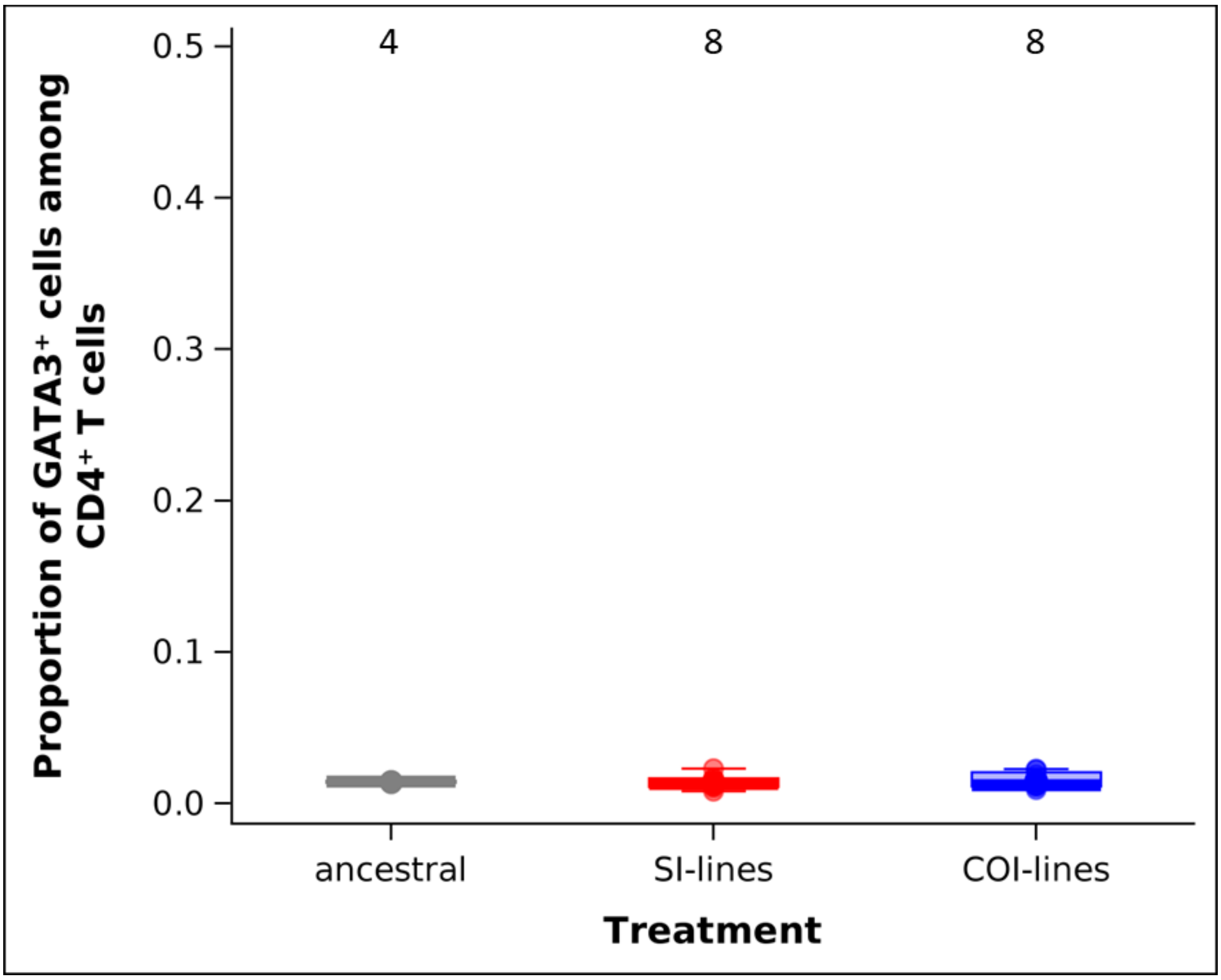

Figure S5. Proportion of GATA-3$^+$ cells after *in vitro* exposure of naïve CD4$^+$ T lymphocytes to HES collected from worms of the ancestral population, SI-lines or COI-lines. The proportion of GATA-3$^+$ cells did not differ between any of the groups (all Bonferroni adjusted p = 1). Dots represent individual observations; boxes represent the interquartile range (IQR); the horizontal line within each box indicates the median; the larger dot indicates the mean; whiskers extend to the most extreme values within 1.5 × IQR from the quartiles; and points beyond the whiskers indicate outliers. Numbers above the boxes indicate the sample size.
